# Multiphoton harvesting metal–organic frameworks

**DOI:** 10.1038/ncomms8954

**Published:** 2015-08-06

**Authors:** Hong Sheng Quah, Weiqiang Chen, Martin K. Schreyer, Hui Yang, Ming Wah Wong, Wei Ji, Jagadese J. Vittal

**Affiliations:** 1 National University of Singapore, Graduate School for Integrative Sciences and Engineering, Singapore 119077, Singapore; 2 Department of Chemistry, National University of Singapore, 3, Science Drive 3, Singapore 117542, Singapore; 3 Department of Physics, National University of Singapore, 3, Science Drive 3, Singapore 117542, Singapore; 4 Institute of Chemical and Engineering Sciences, A*STAR, Jurong Island, Singapore 627833, Singapore

## Abstract

Multiphoton upconversion is a process where two or more photons are absorbed simultaneously to excite an electron to an excited state and, subsequently, the relaxation of electron gives rise to the emission of a photon with frequency greater than those of the absorbed photons. Materials possessing such property attracted attention due to applications in biological imaging, photodynamic therapy, three-dimensional optical data storage, frequency-upconverted lasing and optical power limiting. Here we report four-photon upconversion in metal–organic frameworks containing the ligand, *trans*, *trans*-9,10-bis(4-pyridylethenyl)anthracene. The ligand has a symmetrical acceptor–π–donor–π–acceptor structure and a singlet biradical electronic ground state, which boosted its multiphoton absorption cross-sections. We demonstrate that the upconversion efficiency can be enhanced by Förster resonance energy transfer within host–guest metal–organic frameworks consisting of encapsulated high quantum yielding guest molecules. Using these strategies, metal–organic framework materials, which can exhibit frequency-upconverted photoluminescence excited by simultaneous multiphoton absorption, can be rationally designed and synthesized.

## Abstract (web summary)

Metal–organic frameworks (MOFs) combining the properties of the metal ions and organic ligands are been proposed for many applications. Here Quah *et al*. demonstrate multiphoton excitation fluorescence in MOF materials enhanced by high quantum yielding guest molecules and Förster resonance energy transfer.

## Graphical abstract

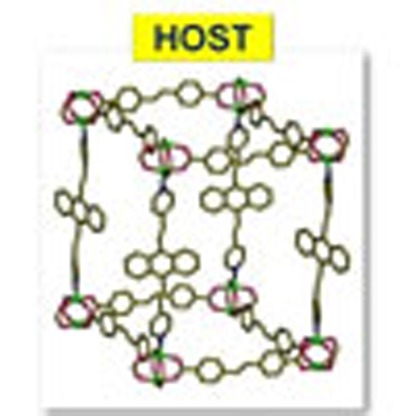

In recent times, materials based on metal–organic frameworks (MOFs)^1^ have been extensively investigated as they hold the key for promising hybrid materials with unique properties. By taking advantage of the combined properties of the metal ions that connect the organic ligands, many functional materials suitable for gas sorption^2^, separation^3^, luminescence^4^, catalysis^5^, magnetism^6^ and conductivity^7^ can be constructed. Herein, we describe a new, previously unexplored feature of MOFs. Multiphoton harvesting (up to five photons) arose from the simultaneous absorption of multiple, identical and energized photons was observed for (*E*)-3-(4-(2-(1-hexyl-4-methyl-1*H*-imidazol-5-yl)vinyl)pyridinium-1-yl)propyl sulphate recently^8^. This is particularly an enticing property suitable for many diverse applications in optical limiting^9^, lasing^10^, optical data storage^11^ and high contrast bioimaging^12^. Compared with the lower-order nonlinear absorption, the fluorescence excited by simultaneous four-photon absorption (4PA) in the short infrared wavelength would possess stronger spatial confinement, larger penetration depth, improved sensitivity and resolution. There have been reports on frequency-upconverted fluorescence from lanthanides^13^,^14^ and lanthanides-based MOFs^15^. However, the absorption and consequential photoluminescence response did not originate from the ligand in the framework but the lanthanide cation, thus restricting amenability to the metal ion. Furthermore, the lanthanide MOFs exhibit sequential stepwise multiphoton excitation but not simultaneous multiphoton absorption that enables higher contrast in bioimaging. By encapsulating suitable dyes in the solvent accessible void of MOFs, two-photon imaging^16^ and lasing^17^ were documented not long ago.

Despite reports on two-photon-excited photoluminescence in zinc(II)-based metal–organic polymers^18^,^19^, the lack of solid-state structure and the difficulty in synthesizing the organic polymer constrained its tunability. In all, the predictability of structural property relationship in MOFs has drawn us to work on this project. It is important to differentiate this work from the second-harmonic generation reported for MOFs^20^. During the revision of this manuscript, Qian *et al*.^21^ reported the incorporation of a zwitterionic ligand into a MOF, which displayed two-photon-excited fluorescence via the multivariate strategy. To rationally synthesize MOFs with multiphoton harvesting property, we select the ligand based on well-established guiding principles in the architecting of chromophoric ligands with large absorption cross-sections (*σ*_*n*_). We successfully synthesized *trans*,*trans*-9, 10-bis (4-pyridylethenyl) anthracene (An2Py) ligand according to literature (see SI for details) and translated its multiphoton harvesting character into three MOFs. The choice of this organic ligand is based on the correlation between nonlinear optical (NLO) response and second hyperpolarizability (**γ**). It has been shown that hyperpolarizability of organic compounds is dependent on several factors such as symmetrical conjugated substituted structure with general structure^22^ of donor–π–acceptor–π–donor (D–π–A–π–D) or acceptor–π–donor–π–acceptor (A–π–D–π–A) arrangement and a biradical ground state that can enhance **γ** (refs 23, 24, 25, 26). Furthermore, models on biradical systems supported that chromophores with a singlet biradical nature^27^,^28^,^29^ can contribute to an enhanced third-order NLO response. Hydrocarbons with an open-shell structure have also been demonstrated to yield an enhancement over the close-shell counterpart^30^.

Here we successfully synthesized a MOF with multiphoton upconversion property (up to simultaneous absorption of four photons at 1,500 nm) by incorporating NLO active organic chromophores as ligands. We demonstrate that the solid-state multiphoton-excited photoluminescence (MEPL) of the MOF is enhanced compared with its organic ligand due to the rigidifying effect in the MOF. Moreover, we achieve further enhancement of the MEPL by encapsulating high quantum yielding guest molecules into the voids of the MOF, providing an additional synthetic possibility to amend the upconversion property. Such enhancement is believed to be caused by the Förster resonance energy transfer (FRET) between the host MOF and the guest molecules. Our initial results suggest that utilizing the above two strategies could make MOF a promising efficient frequency-upconverted luminescent material in the solid state.

## Results

### Synthesis and solid-state structures

**Figure 1:**
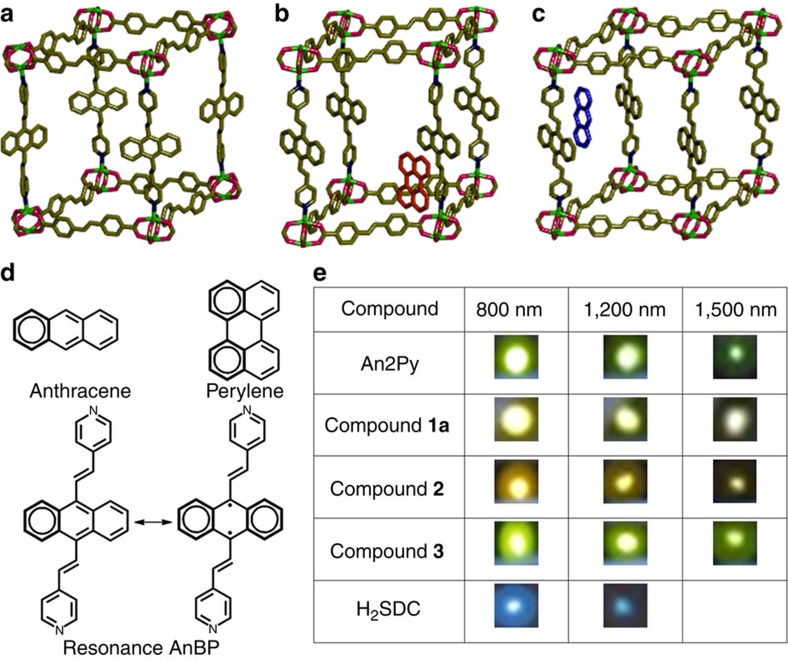
Structures of 1–3, ligands and their fluorescence images. (**a**) A view of **1** showing without interpenetration. Solvents are omitted; (**b**) A view of **2** with encapsulated perylene (orange) guest; (**c**) a view of **3** with encapsulated anthracene (blue) guest; (**d**) Kekulé structures of anthracene, perylene and An2Py (resonance showing possible stabilized biradical structure); and (**e**) luminescence photos of An2Py, **1a**, **2**, **3** at 800, 1200, and 1500, nm femtosecond pulsed laser excitation.

The systematic solvothermal synthesis of three isostructural zinc(II) MOFs with An2Py, *trans,trans*-4,4′ stilbenedicarboxylic acid (H_2_SDC) and two guest molecules is carried out yielding [Zn_2_(SDC)_2_(An2Py)]·dimethyl formamide (DMF)·4H_2_O (**1**), [Zn_2_(SDC)_2_(An2Py)]·perylene (**2**) and [Zn_2_(SDC)_2_(An2Py)]·anthracene (**3**). Solid-state structures of **1**–**3** were determined by single crystal X-ray diffraction techniques and their purities confirmed by powder X-ray diffraction patterns (Supplementary Figs 1–4). Single crystal X-ray diffraction studies revealed that **1** crystallized in the monoclinic space group *C*2/*c* with *Z*=4, whereas **2** and **3** crystallized in monoclinic space group *P*2_1_/*n* with *Z*=2. Nonetheless, all the three MOFs shared the same fourfold interpenetrating **pcu** topology rendering them essentially analogous (Fig. 1a–c; (Supplementary Fig. 5). Their asymmetric units contain half the formula units and crystallographic inversion centres are present in the [Zn_2_(SDC)_2_] paddlewheel units and An2Py ligands. Each paddlewheel repeating unit is shared by four SDC ligands forming a (4,4) net in the (ī01) plane. Each paddlewheel unit is further connected to each other by An2Py pillar ligands. **1** is assembled such that the anthracene moiety of An2Py is stacked alternating along the *c* axis with the closest edge to edge C28–C28 distance between the anthracenes to be 4.55 Å. As for **2**, the tilting and π-stacking are similar to **3**, but differing slightly from the vinyl C7 of the ligand to the centroid of the perylene guest distance, 3.48 Å. The anthracene guest molecules in **3** are π-stacked in between the An2Py along *a* axis and are tilted in the direction of (101) plane with a distance of 3.51 Å from the vinyl C23 of the ligand to the centroid of the guest. Topological study revealed that **3** is slightly compressed as compared with **1**. This is reflected in the diagonal distances of the (4,4) plane (Zṅ̇̇Zn, 27.43 and 20.78 Å in **1** and 30.48 and 16.02 Å in **3**).

### Framework compression due to desolvation

On removal of the guest solvents, **1** does not retain single crystallinity and it slowly became powdery even on exposure to air. But the framework is still intact as the resultant powder remained insoluble in dimethylsulphoxide. To further probe the structure, the guest solvents were fully removed from the framework by heating at 100 °C for 2 h to obtain **1a** and powder X-ray diffraction pattern revealed the formation of a new crystalline phase. This new phase, **1a** is similar to that of the compressed framework of **3** on comparison. It is likely that such flexible paddlewheel framework undergoes contraction^31^ during the solvent removal. Hence, we simulate the crystal structure of the compressed framework, **1a** by removing the anthracene guest and lowering the symmetry of crystal structure **3** to Pī. Using total pattern analysis solution (TOPAS) (Supplementary Fig. 2), the powder data were refined to reveal a new phase ascertaining the phase compression. Despite attempts to obtain 100% framework compressed phase, the framework absorbs moisture from the air to regain partially the original framework of **1**. It was found that the powder used for subsequent analysis consists of 92.23% of the compressed phase (**1a**) and 7.77% of the original uncompressed phase (**1**). For subsequent measurements of multiphoton-excited fluorescence, this dehydrated compound (**1a**) was used for consistency.

### Emission properties by one-photon excitation

**Figure 2:**
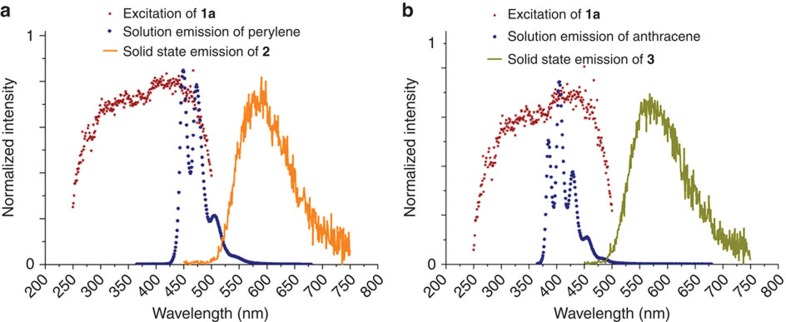
Emission spectra of the guests and MOFs 2, 3 together with the excitation spectra parent MOF 1a showing the occurrence of FRET. (**a**) The emission spectra of the perylene dye coincides with the excitation region of the parent **1a** leading to an emission at 570 nm. The emission of perylene is not observed in **2**. (**b**) The emission spectra of the anthracene dye coincide with the excitation region of the parent **1a** leading to an emission at 570 nm. The emission of anthracene is not observed in **3**.

The selective encapsulation of highly emissive perylene^32^ and anthracene^33^ in **1** resulted in **2** and **3**. These guest molecules have emissions in the excitation region of **1**, which makes FRET^34^ possible (Fig. 2). Furthermore, these aromatic hydrocarbons were also reported to have two-photon excitation fluorescence^35^.

The one-photon-excited photoluminescence studies were carried out before probing the MEPL (Supplementary Fig. 6). At 400-nm excitation, chloroform solution of An2Py displayed fluorescence at 550 nm with quantum efficiency of 35.7% (Supplementary Fig. 7), while H_2_SDC afford significantly lesser, 4.3%. The solid-state emission spectra of compound **1a** revealed higher quantum efficiency (17.3%) compared with powdered An2Py (6.2 %) (Supplementary Fig. 7). This is due to the increased rigidity sustained by the MOF that minimizes non-radiative decay rate due to aggregation induced quenching of the ligand, contributing to the enhanced fluorescence over the ligand itself. Moreover, the MOF emission profile is similar to An2Py (Supplementary Fig. 8). As the ultraviolet absorption of An2Py and the emission of H_2_SDC coincide, it is likely that strut-to-strut^36^ energy transfer has occurred within the MOF leading similar ultraviolet spectra (Supplementary Fig. 9). The quantum yield of **2** (25.4%) and **3** (26.2%) measured were found to be much better than **1a**. The π–π stacking of the anthracene guest molecules (perylene in **2** and anthracene in **3**) and An2Py brought them within close proximity at which FRET is allowed to occur. Furthermore, the spectra overlap of the guest emission and the excitation of the MOF suggested FRET occurred (Fig. 2). As a result, the emission of the guest molecules were quenched in **2** and **3** on excitation and a sole broad fluorescence band of 570 nm appeared, validating the energy transfer that led to the emission enhancement.

### Multiphoton excitation photoluminescence

Femtosecond laser pulses (150 fs, 1 kHz repetition rate) with wavelengths ranging from 800 to 1,500 nm were subsequently used to study the MEPL at room temperature. The measurements were carried out by packing well powdered single crystals of the ligands and MOFs in a 1-mm-thick quartz cuvette (Supplementary Figs 10 and 11). Multiphoton action cross-section, which is the product of multiphoton absorption cross-section (*σ*_*n*_) and photoluminescence quantum yield (*η*), is a direct measurement of the MEPL brightness. To determine the *σ*_*n*_ values of the investigated samples, perylene, which shows two-photon emission on exciting with 800-nm laser beam, was utilized as a standard sample^35^,^37^.

**Figure 3:**
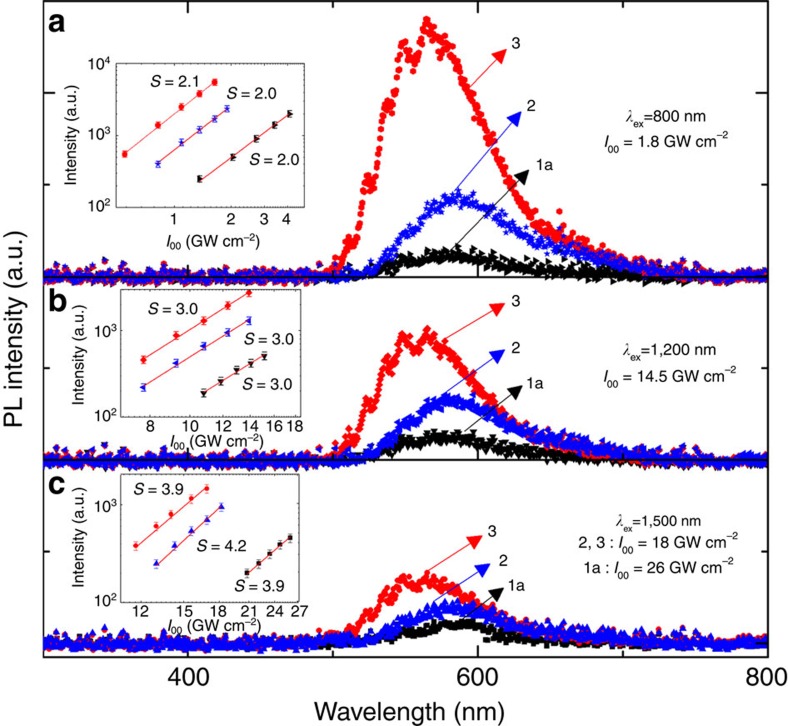
Photoluminescence spectra of three MOFs in the powder form with femtosecond pulsed laser excitation. The inset shows the dependence on laser intensity. (**a**) Two-photon-excited emission at 800 nm of **1a**–**3**. (**b**) Three-photon-excited emission at 1,200 nm of **1a**–**3**. (**c**) Four-photon-excited emission at 1,500 nm of **1a**–**3**.

Photos in Fig. 1e clearly demonstrate the frequency upconverted photoluminescence when the MOFs are illuminated with near-infrared femtosecond laser pulses. The solid-state reflectance spectra of the compounds indicated absorption maxima at 400 nm (Supplementary Fig. 9). Correspondingly, the MEPL spectra of the MOF samples collected at 800 nm for two-photon absorption (2PA), 1,200 nm for three-photon absorption (3PA) and 1,500 nm for four-photon absorption (4PA) are displayed in Fig. 3. (Note: different samples have different molar concentration used for the determination of *σ*_*n*_*η* as recorded in the Supplementary Table 1.) Excitation intensity dependence of the MEPL signal (shown in the inset of Fig. 3) further justify that 2PA, 3PA and 4PA are responsible for the excitation at 800, 1,200 and 1,500 nm, respectively. The same photoluminescence spectra profile obtained regardless of a single-photon exitation, or multiphoton excitation (Supplementary Fig. 12), is indicative that the emission involves the same electronic state of the MOFs.

**Figure 4:**
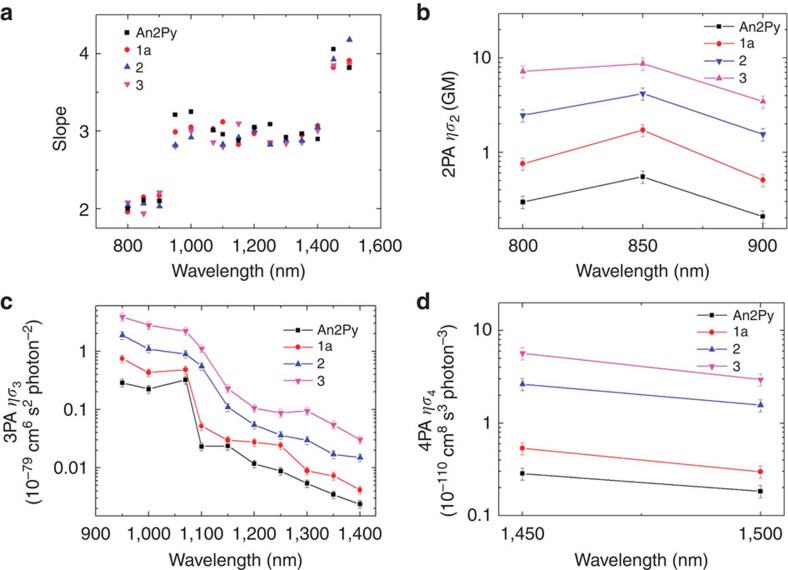
Multiphoton-excited fluorescence action cross-sections for An2Py, 1a, 2 and 3. (**a**) Slopes *n* plotted as a function of laser excitation wavelength, where *n* is defined by the excitation intensity dependence of photoluminescence signal that is proportional to (excitation intensity)^*n*^; (**b**) two-photon action cross-sections of An2Py, **1a**, **2**, **3**; (**c**) three-photon action cross-sections of An2Py, **1a**, **2**, **3**; and (**d**) four-photon action cross-sections of An2Py, **1a**, **2**, **3**. Here, the experimental error results mainly from the uncertainty in fluctuation of input laser pulse energy and determination of laser beam characteristics such as beam waist and pulse duration. As shown in equation (1) and also expressions of *F*_2_, *F*_3_ and *F*_4_ in Supplementary Methods, 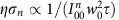, and 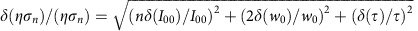. *I*_00_ is laser beam peak intensity, *w*_0_ is the beam waist and *τ* is the laser pulse duration

Furthermore, the slopes indicative of the order of absorption process from the excitation intensity dependence of MEPL signals (An2Py and three MOFs) across different wavelengths were summarized in Fig. 4a. In the short-wavelength range from 800 to 900 nm, the slopes of photoluminescence from the samples are around 2, clearly indicating 2PA. 3PA processes with slopes increasing to around 3 appear to dominate as the excitation wavelength is increased from 950 to 1,400 nm. As excitation wavelengths are increased to the range from 1,450 to 1,500 nm, the slopes are around 4, signalling the dominance of 4PA processes.

As for the co-ligand H_2_SDC, its one-photon absorption peak at around 384 nm. As such, the wavelength range in which the *n*-photon absorption process occurs differs from An2Py and the MOFs. 3PA fluorescence is dominant in the wavelength range of 800–1,150 nm with slope 3. While at longer wavelengths of 1,200–1,350 nm, a 4PA fluorescence process occurred (Supplementary Fig. 11).

### Multiphoton action cross-section of the MOFs

The MEPL strength *F*_*n*_ can be quantify and obtained by integrating Δ*f*_*n*_ given by equation (1) over the entire laser focused volume and time^38^,^39^,^40^.


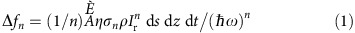


The factor of 1/*n* accounts for the fact that *n*-photons are simultaneously absorbed from the near-infrared laser light for each fluorophore excitation generated. *ħω* is the photon energy of the near-infrared incident laser beam. Ø is the fluorescence collection efficiency of the experimental setup, *ρ* is the sample molar concentration and *I*_r_ is the nearly constant laser intensity at this small volume. With several reasonable assumptions, the strength of the collected MEPL signal (*F*_*n*_) can be obtained for 2PA, 3PA and 4PA processes, respectively. (Supplementary Figs 10 and 11)

Utilizing perylene as a standard sample, the *ησ*_*n*_ (*n*=*n*th order photon absorption) values of the An2Py and MOFs were determined and shown in Fig. 4b–d, respectively. Here the experimental error results mainly from the uncertainty in fluctuation of input laser pulse energy and determination of laser beam characteristics such as minimum beam waist and pulse duration.

## Discussion

To demonstrate the amenability of the *σ*_*n*_ (*n*=*n*th order photon absorption) in MOFs, the selective encapsulation of highly emissive perylene and anthracene in **1** was carried out, which resulted in **2** and **3**. These guest molecules have high quantum efficiency and an emission in the excitation region of **1** that makes FRET possible (Fig. 2). The π–π interactions between the ligand and guest ensured the ease of host–guest formation during the synthesis without altering the interpenetration or topology. In addition, such aromatic hydrocarbons were reported previously to have two-photon excitation fluorescence. The same photoluminescence spectra profile obtained regardless of a single-photon excitation, or multiphoton excitation (Supplementary Fig. 12), is indicative that the emission involves the same electronic state of the MOFs.

Within the region of 2PA process, the measured *ησ*_2_ are in the range of 0.2–7.2 GM with a maximum at 850 nm for all the samples. As depicted in Fig. 4b, **1**a has larger 2PA action cross-section than An2Py, which is about three times greater. This difference can be mainly attributed to **1a** possessing about three times higher fluorescence quantum yield (17.3%) than An2Py (6.2%), as a result of the increased rigidity sustained by MOF. This trend is similar to the linear excited fluorescence. Enhanced *ησ*_2_ observed in **2** and **3** came from FRET occurring between the encapsulated guests and the host MOF. Overall, the measured *ησ*_2_ is relatively small compared with the usual solution samples expected from laser dyes. This can be due to reabsorption and scattering effects of solid samples that is present during the collection process. In the wavelength range of the dominant 3PA process (950–1400, nm), a maximum *ησ*_3_ at 950 nm was revealed for all the samples and it decreases with increasing wavelength. The maximum *ησ*_3_ obtained among the compounds is (3.9±0.6) × 10^−79^ cm^6^ s^2^ per photon^2^ for **3** at 950 nm. However, similar to 2PA, the *ησ*_3_ value could be underestimated with the measurement setup. Noteworthyly, this behaviour of *ησ*_3_ decreasing with the increasing wavelength is similar to typical semiconductor crystals. It is documented that 3PA coefficient is a function of the photon energy as (3*ħω*/*E*_g_–1)^5/2^(3*ħω*/*E*_g_)^−9^, where *E*_g_ is the bandgap for the semiconductor crystals^41^. Strikingly, although **1a** has almost the same *σ*_3_ as that of An2Py, it demonstrates thrice as large fluorescence (*ησ*_3_) than An2Py due to its three times higher fluorescence quantum yield (*η*). On a separate note, similar enhancements in *ησ*_3_ were obtained in **2** and **3** as a result of FRET between the host MOF and guest molecules perylene or anthracene. Four-photon process is further demonstrated at 1,450 and 1,500 nm, and similar trend to that of 2PA and 3PA were observed. Although it is underestimated, the maximum *ησ*_4_ obtained among the compounds is (5.7±0.8) × 10^−110^ cm^8^ s^3^ per photon^3^ for **3** at 1,450 nm. Across all wavelengths, it is evident (Fig. 4) that **3** possessed the highest multiphoton action cross-section among the MOFs synthesized. This could be the better overlap of the anthracene emission with the MOF compared with the perylene guest. Nevertheless, **2** exhibited higher excited fluorescence than **1a** at all wavelengths due to FRET while the enhancement demonstrated by all the MOFs over the ligand is due to the rigidity sustained by the MOF framework.

To investigate the open-shell character of the compounds, the solid-state electron paramagnetic resonance (EPR) spectroscopy was used to probe An2Py, H_2_SDC and the three MOFs (Supplementary Figs 13–17) for open-shell character. Indeed, the spectra revealed the presence of an isotropic radical signal with *g*-values at 2.00412, 2.00177, 2.00392, 2.00348 and 2.00424 for An2Py, H_2_SDC, **1a**, **2** and **3**, respectively. However, it is crucial to note that the signal from H_2_SDC is extremely weak compared with An2Py and MOFs. The *g*-values of the compounds are very close to 2.00232, which are for a free electron with no spin coupling. This confirms that the open-shell character of An2Py has been incorporated into the MOFs as they present the same isotropic signal in their EPR spectra. Although both ligands seemed to contribute to the radical electronic state in the MOFs, it appeared that the contribution from An2Py is more significant as judged by the more intense signal. The variable temperature EPR was carried out for An2Py and it showed a decreasing signal with decreasing temperatures (Supplementary Fig. 13b) indicating a singlet biradical ground state that is in equilibrium with a higher energy triplet biradical state^42^.

Subsequently, multiconfiguration calculations (see SI for details) suggested that An2Py should exist as a closed-shell molecule as the most stable ground state derived is the closed-shell configuration. We are able to locate an open-shell singlet state that is 165.8 kJ mol^−1^ higher in energy than a closed-shell singlet state. The triplet open-shell state is only 1.3 kJ mol^−1^ higher in energy than the open-shell singlet state. Interestingly, the chloroform solution of An2Py ligand is EPR silent (Supplementary Fig. 13c) and the ^1^H-NMR spectrum of An2Py ligand in CDCl_3_ solution is normal, neither broadened nor shifted due to paramagnetic nature. These results are consistent with the multireference calculations carried out for the individual molecules. However, the solid-state ligand exhibits an open-shell character contrary to the solution state. Hence, we attribute the behaviour of the ligand in the solid state to packing effect (‘crystal property') as opposed to its molecular behaviour in solution. In the solid state, the crystal close packing often allows intermolecular interactions that lead to a different photoluminescence profile from the solution state. These intermolecular interactions are accountable for lower quantum efficiency, broadening and stoke shifts of the emission profile^43^. As such, the findings of multiconfiguration calculation cannot completely describe An2Py in the solid state but accurately for isolated single molecule. Nonetheless, the solid-state singlet open-shell character of An2Py was experimentally verified by the variable temperature EPR.

In summary, usually multiphoton harvesting property exhibited by the organic molecules in the solution is quenched in the solid state due to aggregation. However, efficiently luminescent materials in the solid-state form are highly preferable in many photonic applications such as upconversion lasing and light emission due to a higher resistant to photobleaching. We demonstrated that MOFs with large NLO response can be designed by rational choice of ligands with high second hyperpolarizability (**γ**). With both A–π–D–π–A structure and open-shell singlet character, An2Py displayed multiphoton-excited fluorescence signal strong enough to be collected and characterized. The property was duly expressed in the MOF that formed. This shows a successful incorporation of a multiphoton-excited emissive organic chromophore into a MOF that resulted in solid-state MEPL. The rigidifying of the chromophore in the MOFs led to the enhancement in MEPL. Subsequent incorporation of suitable guests in the voids of **1a** resulted in MOFs **2** and **3** and displayed enhancement due to FRET providing an additional synthetic possibility to amend the upconversion property.

## Methods

### Materials

Preparation of the ligand An2Py was according to reported procedures (see SI). Starting materials are purchased from Sigma Aldrich Chemicals unless otherwise stated. Perylene and anthracene were obtained from Alfa Aesar.

### Synthesis

Preparation of [Zn_2_(SDC)_2_(An2Py)]·DMF·4H_2_O(**1**): 9.3 mg (0.025 mmol) of Zn(ClO_4_)_2_, 4.8 mg (0.0125, mmol) of An2Py and 6.7 mg (0.025 mmol) of H_2_SDC were weighed in a 25-ml scintillating vial. Two mililitre (ml) of DMF and 1 ml of water were added to the solids with a drop of HNO_3_. The bright red solution that formed in the vial was capped and heated in the oven for 2 days at 120 °C followed by slow cooling. Bright yellow plate-like crystals were obtained and washed with DMF. Yield: 50%

Preparation of [Zn_2_(SDC)_2_(An2Py)](**1a**): (**1**) was ground and heated at 100 °C for 2 h under vacuum. The bright yellow powder was used for further characterization.

Preparation of [Zn_2_(SDC)_2_(An2Py)]·perylene (**2**): 3.15 mg (0.0125, mmol) of perylene was weighed into a 25-ml scintillating vial containing the chemicals from **1**. Two days at 120 °C followed by slow cooling yielded bright orange plate-like crystals. Yield: 53%

Preparation of [Zn_2_(SDC)_2_(An2Py)]·anthracene (**3**): 2.2 mg (0.0125, mmol) of anthracene was weighed into a 25 -ml scintillating vial containing the chemicals from **1**. Two days at 120 °C followed by slow cooling yielded bright yellow plate-like crystals. Yield: 54%

### Optical measurements

One-photon-excited absorption and fluorescence spectra for solution samples were acquired through utilizing an UV-1601 Shimadzu ultraviolet–visible spectrometer and a Varian Cary Eclipse Fluorescence Spectrophotometer, respectively.

One-photon-excited fluorescence spectra for well powdered single crystals were collected by employing a Horiba Jobin-Yvon FluoroMax-4 Spectrofluorometer with appropriate sample holder at excitation wavelength of 400 nm. Excitation spectra of the well powdered single crystals were obtained in the same set-up at their peak emission wavelengths. Quantum yield measurements were performed using the absolute method on a fluorescent spectrometer FSP920 from Edinburgh Instruments equipped with a BaSO_4_-coated integrating sphere, a xenon lamp and a photomultiplier tube detector.

MEPL measurements were conducted using the following apparatus. The excitation laser pulses (1 kHz, 285–2,600 nm, pulse width <150 fs) were generated by an optical parametric amplifier (TOPAS-C, Light-Conversion) pumped by a regenerative amplified femtosecond Ti:Sapphire laser system (800 nm, 1 kHz, pulse energy 3 mJ, pulse width <150 fs, Libra, Coherent), which was seeded by a femtosecond Ti-saphhire oscillator (80 MHz, pulse width <100 fs, 800 nm, Vitesse TM 800-2, Coherent). The MEPL spectra of the excitation pulses were recorded with a spectrometer (Avaspec-2048-SPU, Resolution of 0.5 nm; see SI). The laser power was measured by an optical power meter (Optical power meter 1917-R, Newport) with the appropriate sensor. (Detector 919P-003-10, Newport).

## Additional information

**Accession codes.** CCDC 1041093, 1041094 and 1041092 (**1**, **2** and **3**) contains the supplementary crystallographic data for this paper. These data can be obtained free of charge from the Cambridge Crystallographic Data Centre via www.ccdc.cam.ac.uk/data_request/cif

**How to cite this article:** Quah, H. S. *et al*. Multiphoton harvesting metal–organic frameworks. *Nat. Commun.* 6:7954 doi: 10.1038/ncomms8954 (2015).

## Supplementary Material

Supplementary Information Supplementary Figures 1-28, Supplementary Tables 1-3, Supplementary Discussion, Supplementary Methods and Supplementary References
