# Supplementary material for: Multiphoton harvesting metal–organic frameworks
Source: Nat Commun. 2015 Aug 6;6:7954. doi: 10.1038/ncomms8954 (PMC4918338; doi:10.1038/ncomms8954)
Supplement: Supplementary Information — Supplementary Figures 1-28, Supplementary Tables 1-3, Supplementary Discussion, Supplementary Methods and Supplementary References [file ncomms8954-s1.pdf]

Supplementary Figures

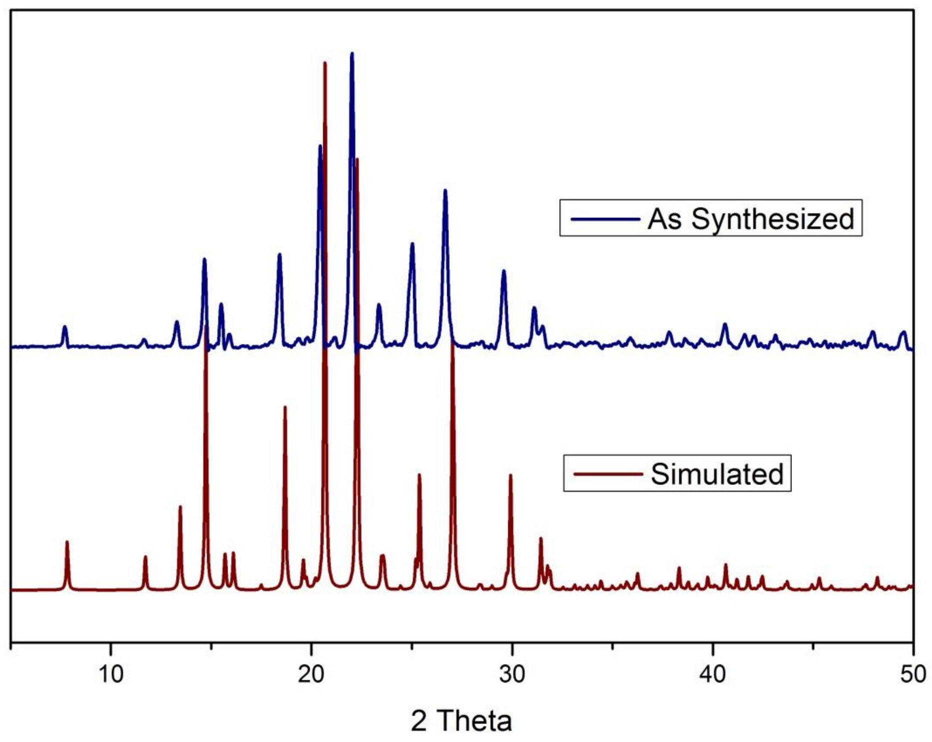

Supplementary Fig. 1: Simulated and bulk PXRD patterns of An2Py ligand.

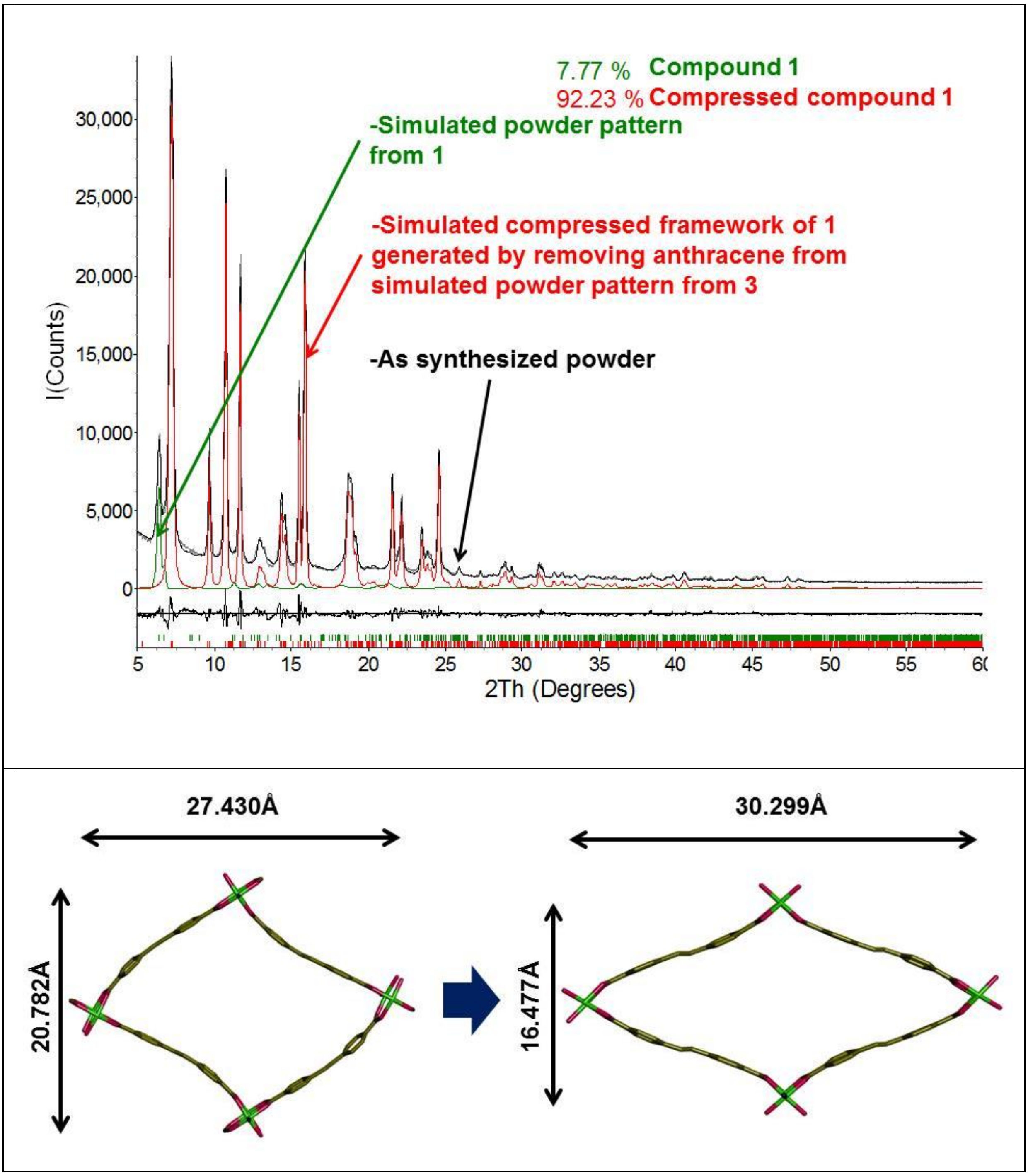

Supplementary Fig. 2: X-ray powder patterns of 1 and 1a, and structure solution by TOPAS. (**TOP**) Structure solution of the desolvated compound **1a** by Rietveld refinements. Despite efforts to fully remove the solvent molecule, the reabsorption of moisture from the atmosphere led to the sample retaining 7.77% of the uncompressed phase. (**Bottom**) The pictorial representation of the framework compression of **1** to **1a** due to desolvation.

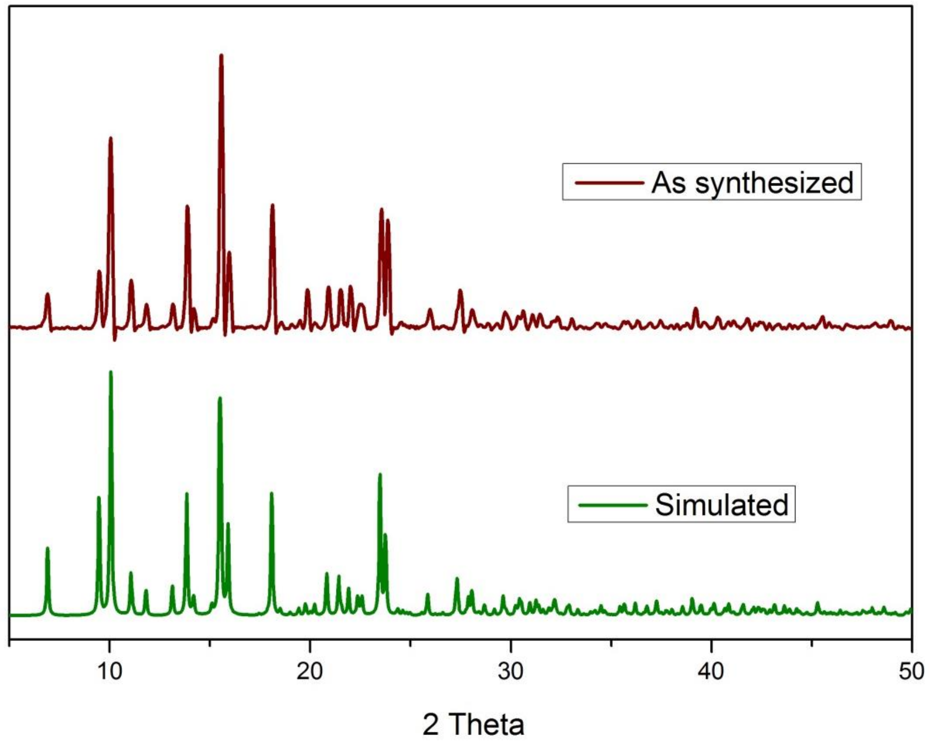

Supplementary Fig. 3: Simulated and bulk PXRD patterns of **2**.

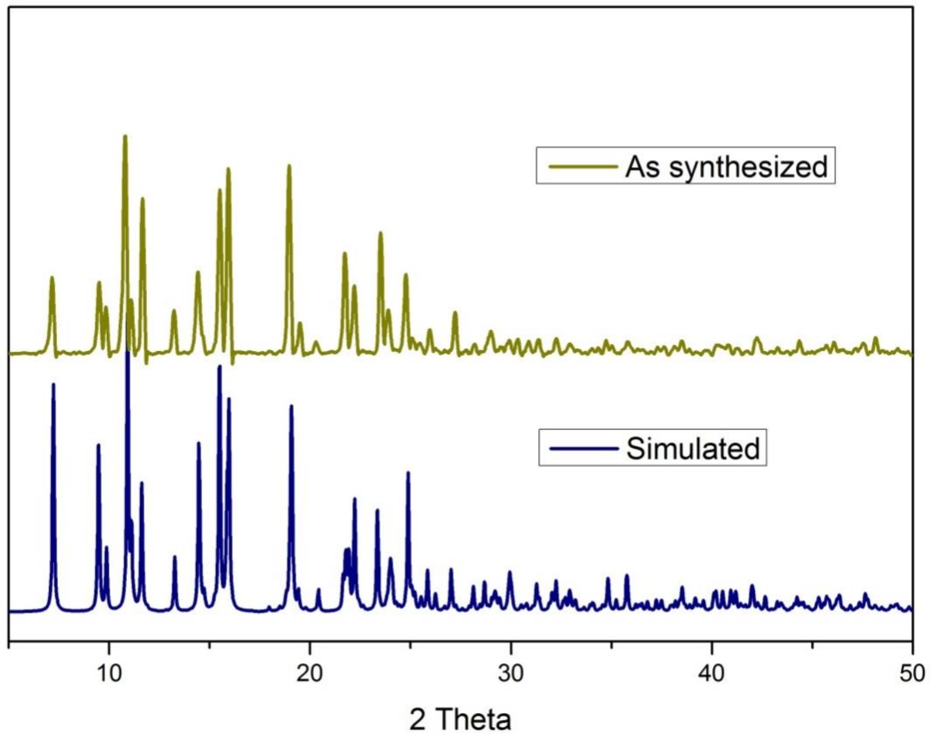

Supplementary Fig. 4: Simulated and bulk PXRD patterns of **3**.

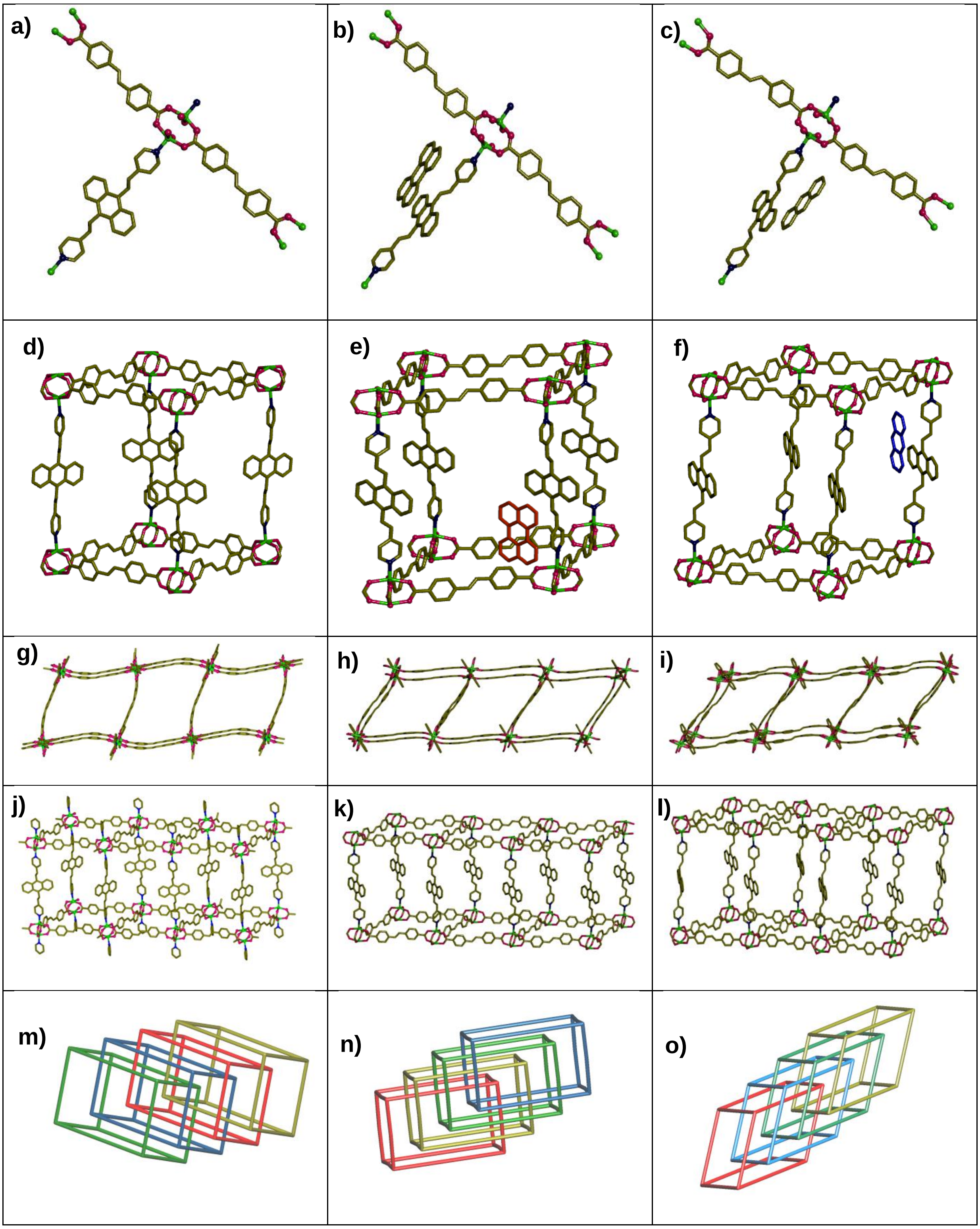

a)
b)
c)
d)
e)
f)
g)
h)
i)
j)
k)
l)
m)
n)
o)

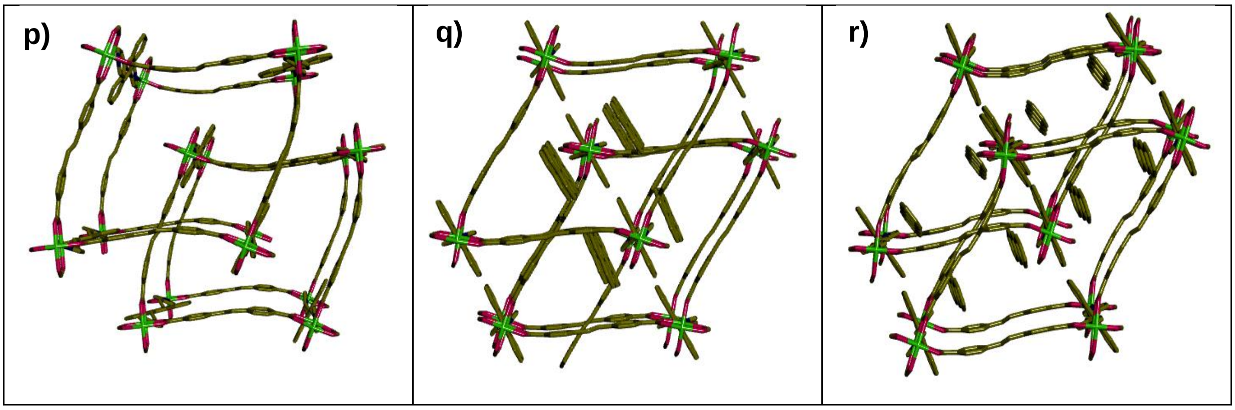

Supplementary Fig. 5: Crystal structure packing. (**a-c**) A view of a portion of **1, 2** and **3**. (**d-f**) pcu packing of **1, 2** and **3**. (**g-i**) Packing of **1-3**. (**j-l**) Alternate views of the packing of **1-3** (**m-o**) Simplified toplogical net of **1-3** showing 4-fold interpenetrating **pcu** nets. (**p-r**) Partial views showing the interpenetrated packing.

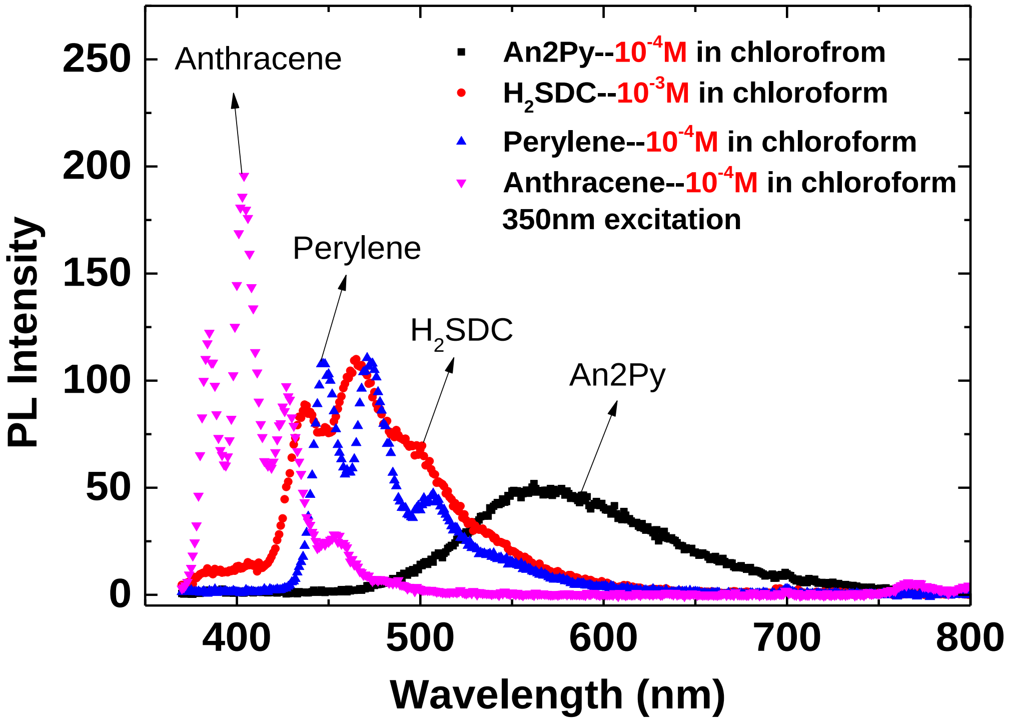

Supplementary Fig. 6: Solution photoluminescence of organic compounds used.

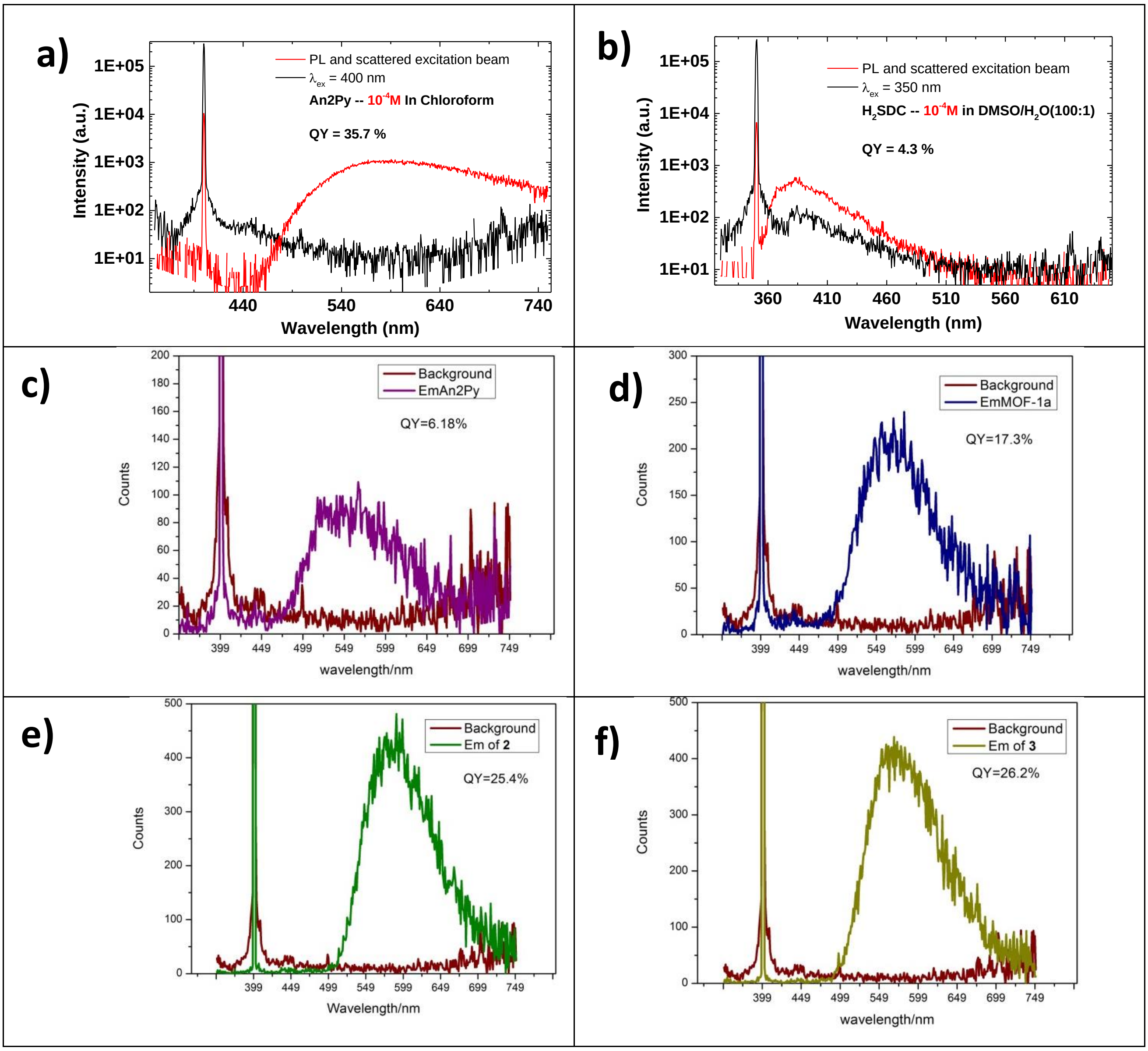

Supplementary Fig. 7: Solid-state PL spectra and absolute quantum efficiency as derived from an integrating sphere. (**a**) Solution quantum efficiency (QE) of An2Py in chloroform (10^{-3} M) (**b**) solution QE of H_2SDC in DMSO/H_2O (10^{-4}M) (**c**) solid state PL spectra and QE of An2Py (**d**) solid state QE of **1a** (**e**) solid state QE of **2** **(f**) solid state QE of **3**.

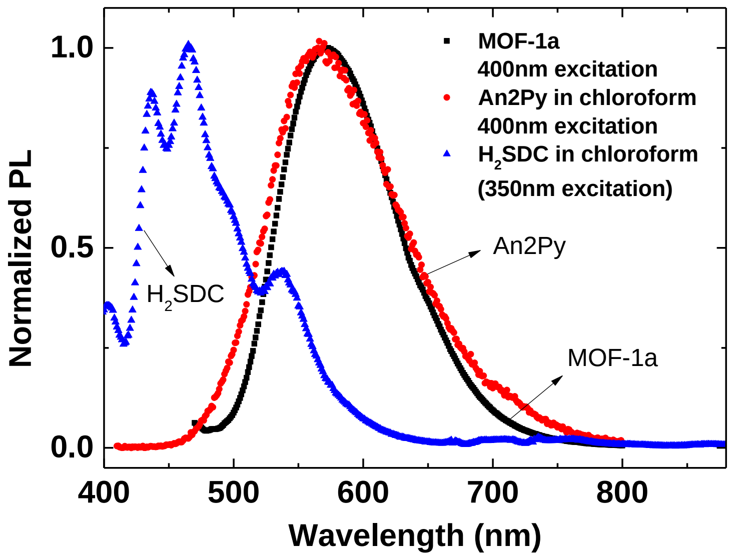

Supplementary Fig. 8: Comparison of the PL spectra of **1a** with those of two ligands An2Py and H_2SDC, revealing that the PL of **1a** mainly originates from one of its ligands, An2Py.

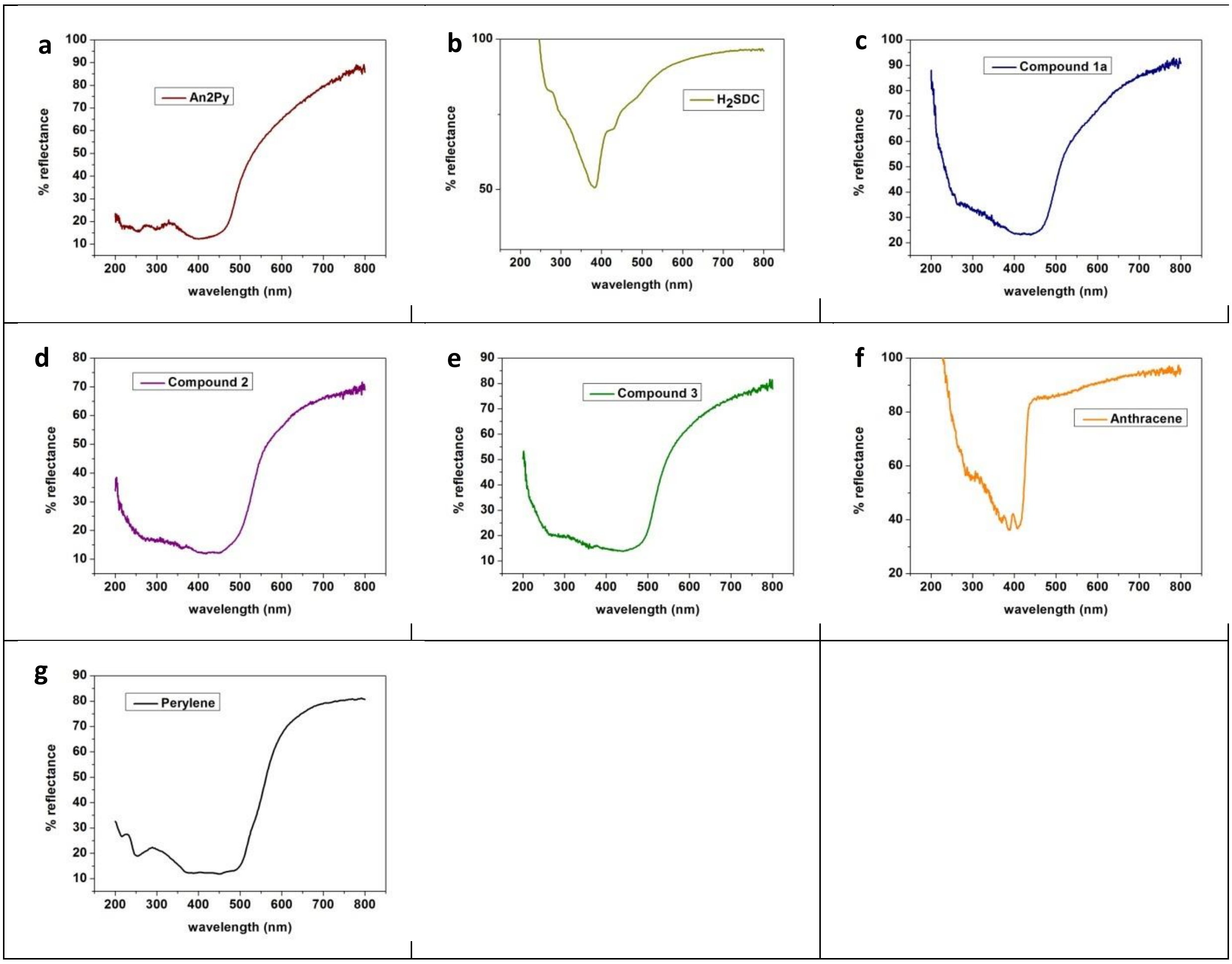

Supplementary Fig. 9: Solid-state diffuse reflectance UV spectra of all compounds used. (**a**) An2Py (**b**) H_2SDC (**c**) Compound 1a (**d**) Compound 2 (**e**) Compound 3 (**f**) anthracene (**g**) perylene.

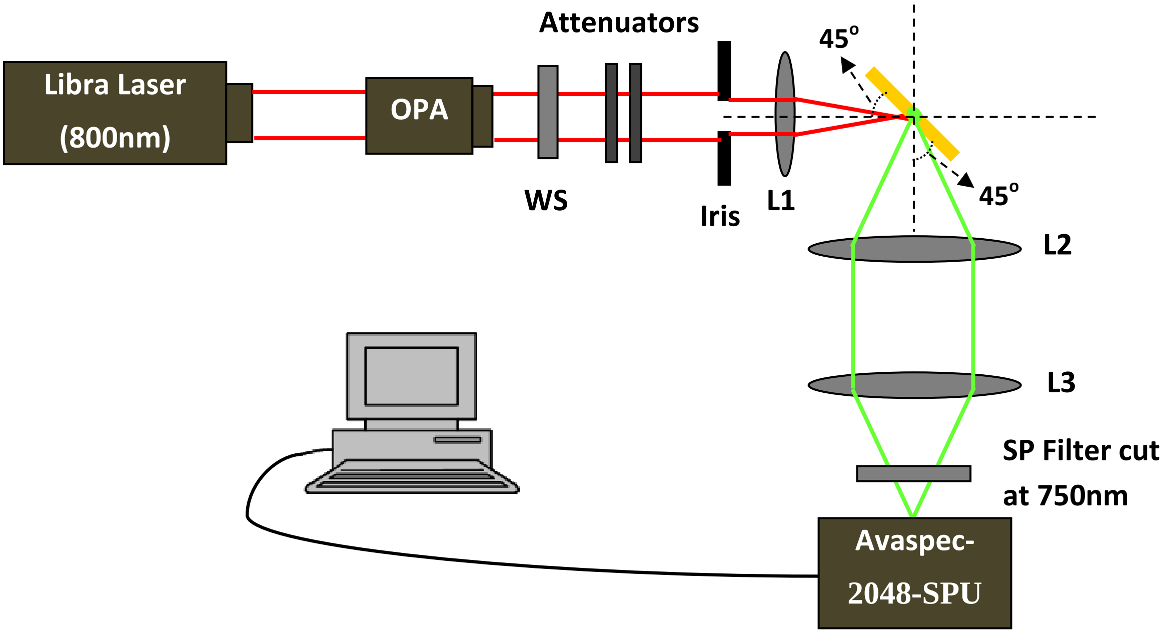

Supplementary Fig. 10: Experimental setup for measuring MEPL. The excitation wavelength was varied between 800 and 1500 nm to allow two-, three- and four-photon absorption excited fluorescence measurements. OPA, WS, and SP are short for optical parametric amplifier, wavelength separators, and short-pass optical filter, respectively.

Compound	λ_{ex} = **800 nm**	λ_{ex} = **1200 nm**	λ_{ex} = **1500 nm**
Compound **1a**	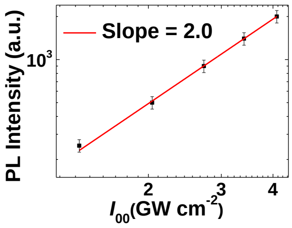	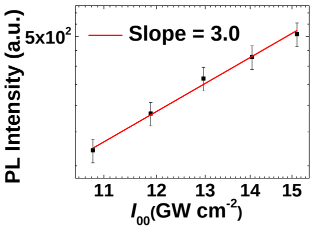	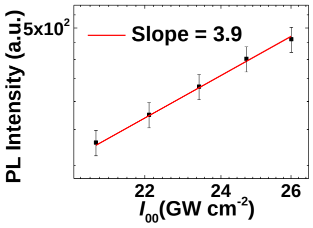
Perylene ⊂Compound **1a** Compound **2**	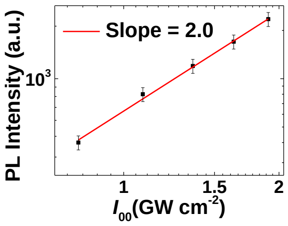	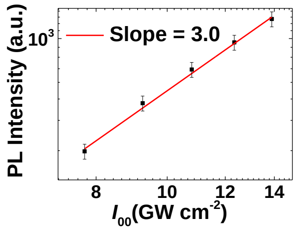	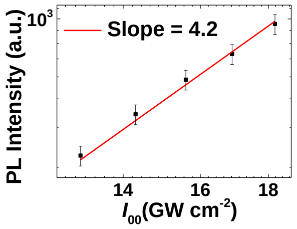

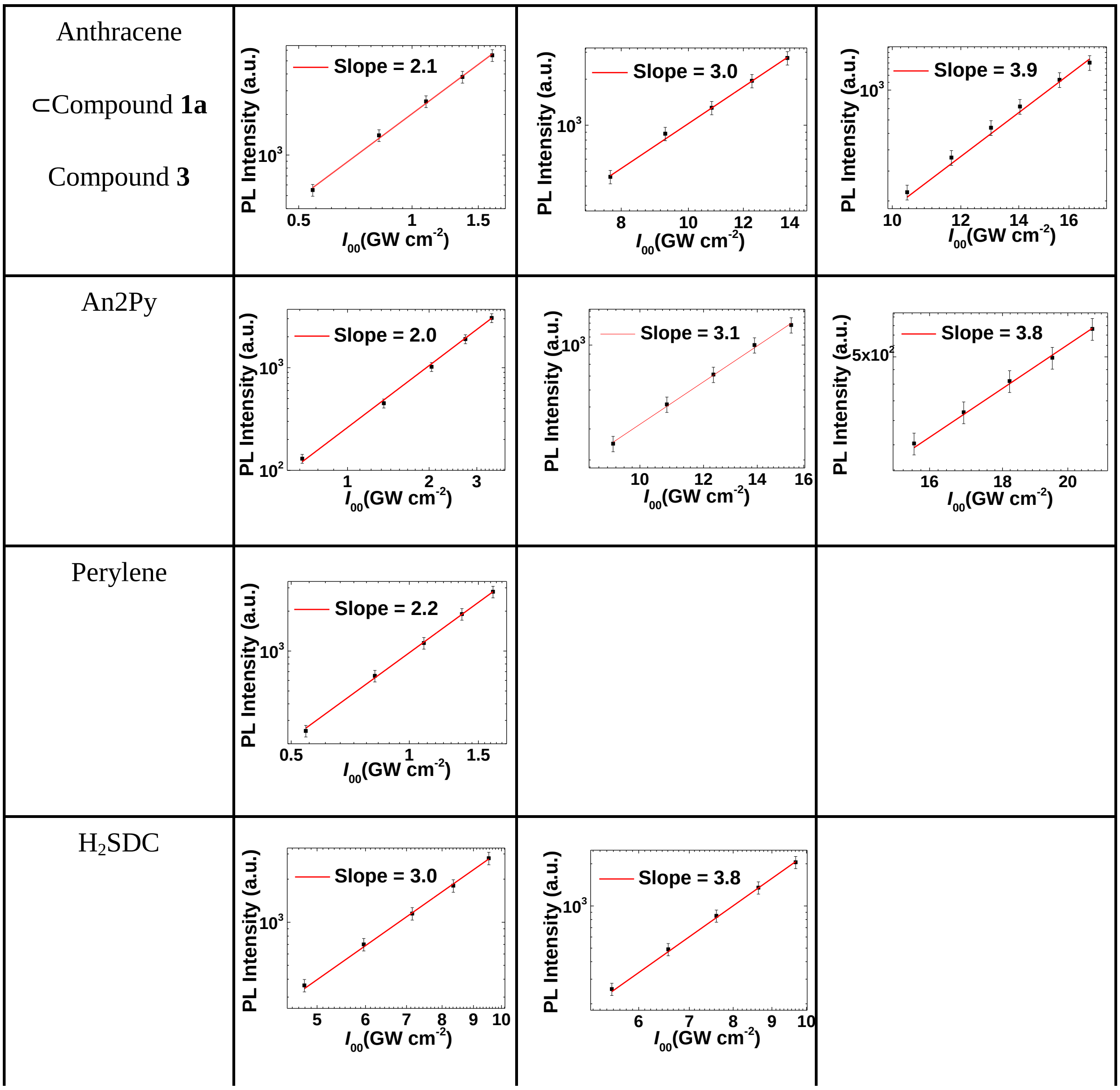

Supplementary Fig. 11: Excitation intensity dependence of MEPL peak intensity for the An2Py, H_2SDC and MOFs at excitation wavelengths of 800, 1200 and 1500 nm. As a calibration, excitation intensity dependence of perylene was measured at 800 nm.

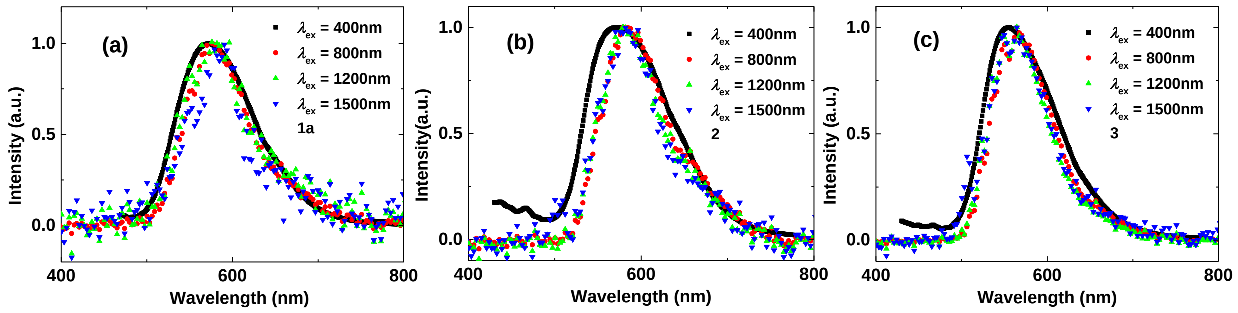

Supplementary Fig. 12: Comparison of one-photon-excited and MEPL spectra of compound **1a**, **2** and **3**.

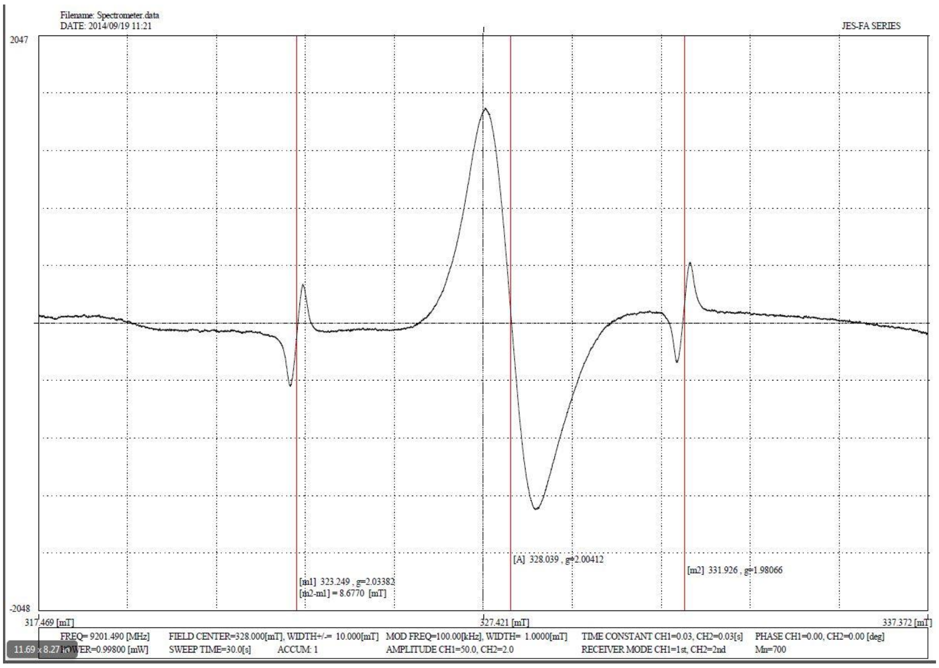

Supplementary Fig. 13a: EPR of An2Py showing an isotropic singlet in the solid state at room temperature with *g* value = 2.00412. The additional peaks on both sides of the spectrum are due to the standard Mn^{2+} marker.

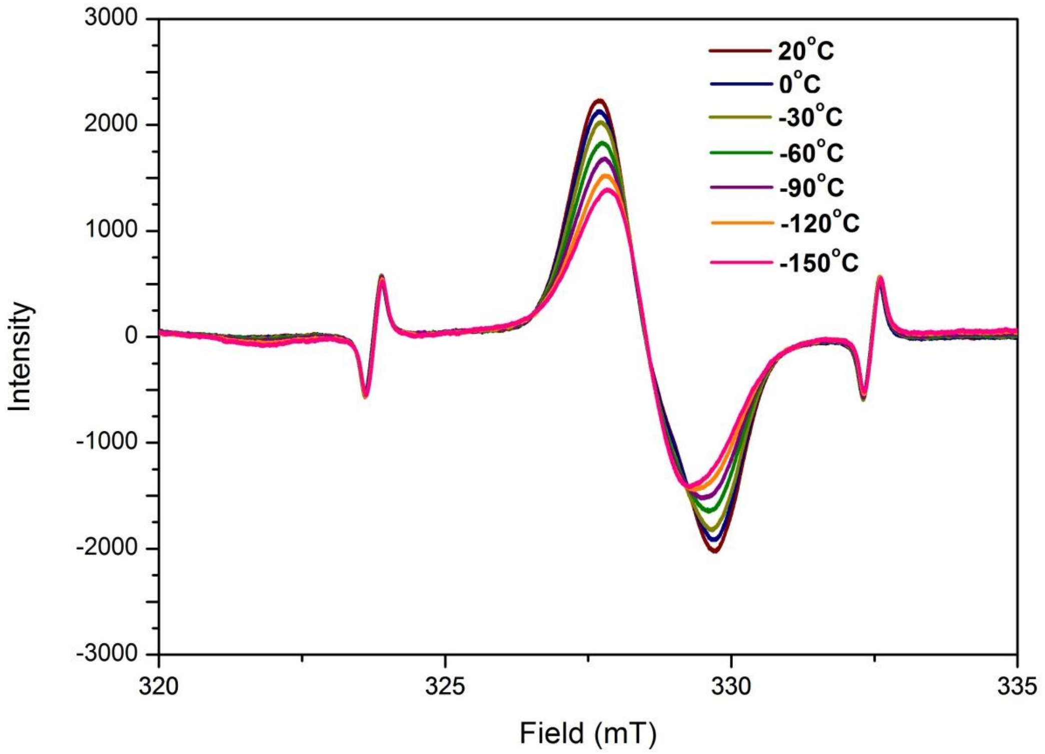

Supplementary Fig. 13b: Variable Temperature EPR (VTEPR) of An2Py displaying a decreasing signal with decreasing temperatures. The additional peaks on both sides of the spectrum are due to the standard Mn^{2+} marker.

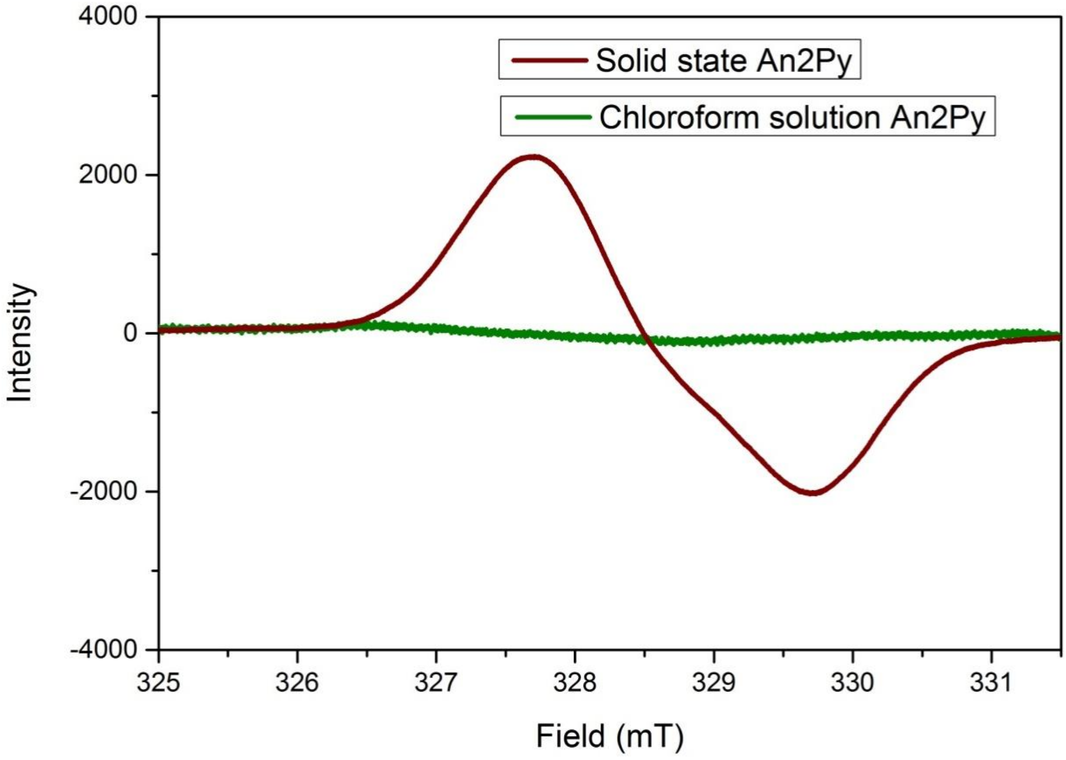

Supplementary Fig. 13c: Solution and solid state EPR signals of An2Py. The chloroform solution EPR of An2Py is silent.

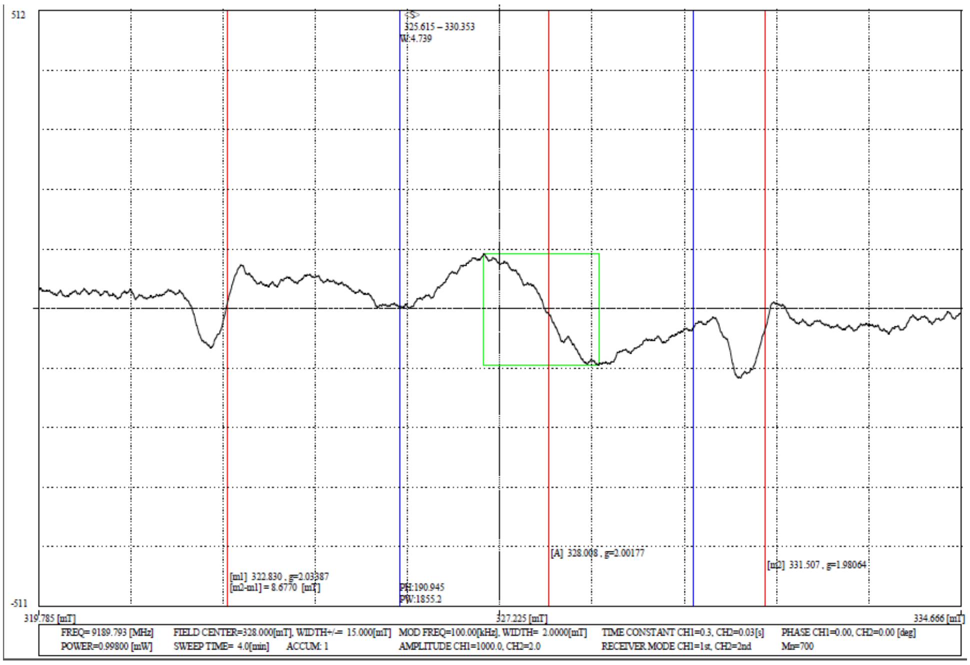

Supplementary Fig. 14a: EPR of H_2SDC showing a weak singlet in the solid state at room temperature with *g* value = 2.00177. The additional peaks on both sides of the spectrum are due to the standard Mn^{2+} marker.

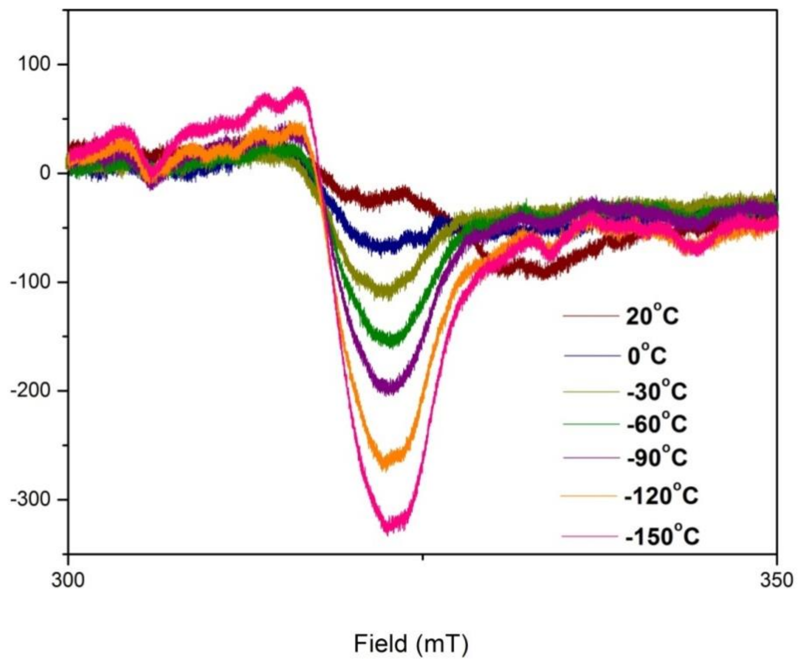

Supplementary Fig. 14b: Variable Temperature EPR (VTEPR) of H_2SDC displaying an extremely weak signal with the waveform deforming with decreasing temperatures.

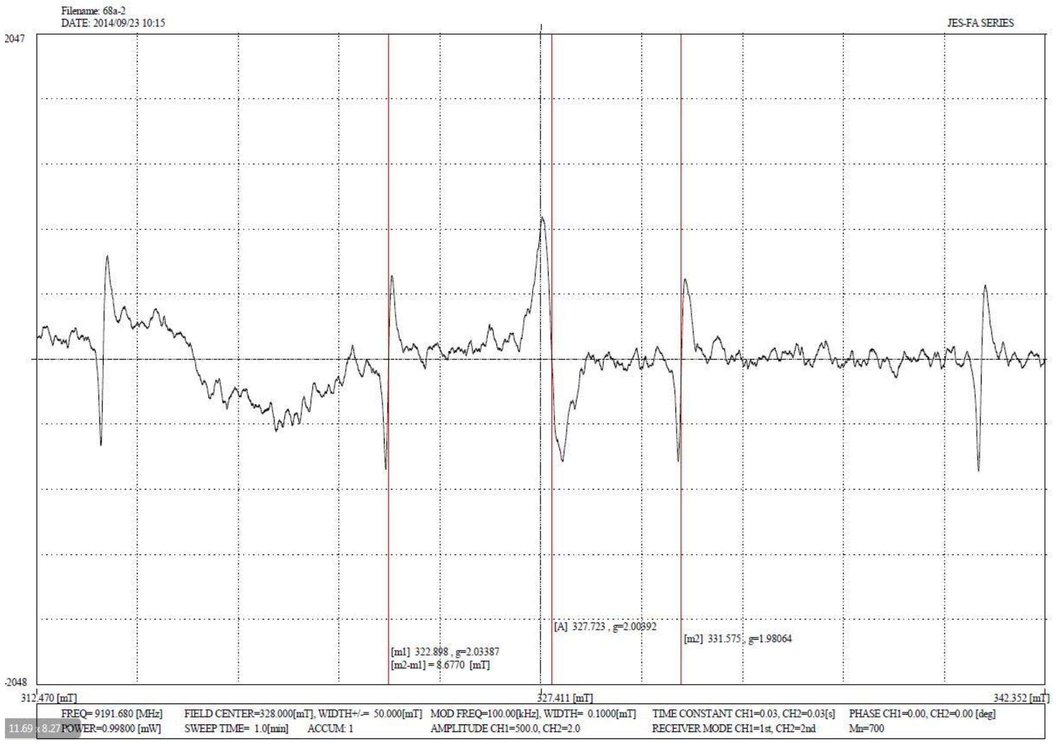

Supplementary Fig. 15: EPR of **1a** showing singlet in the solid state at room temperature with *g* value = 2.00392. The additional peaks on both sides of the spectrum are due to the standard Mn^{2+} marker.

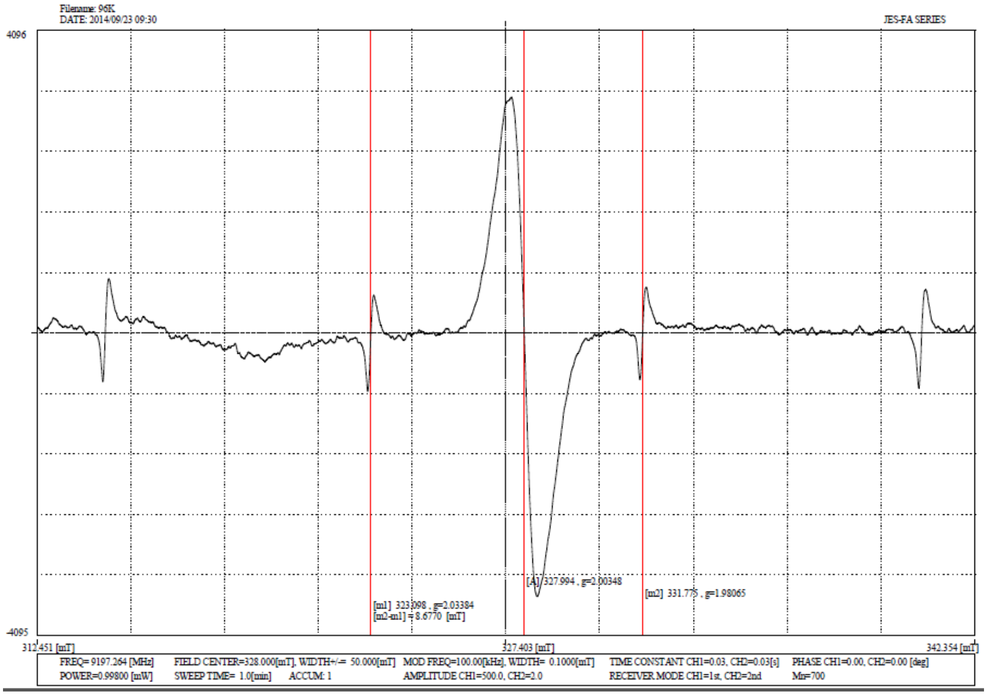

Supplementary Fig. 16: EPR of **2** showing singlet in the solid state at room temperature with *g* value = 2.00348. The additional peaks on both sides of the spectrum are due to the standard Mn^{2+} marker.

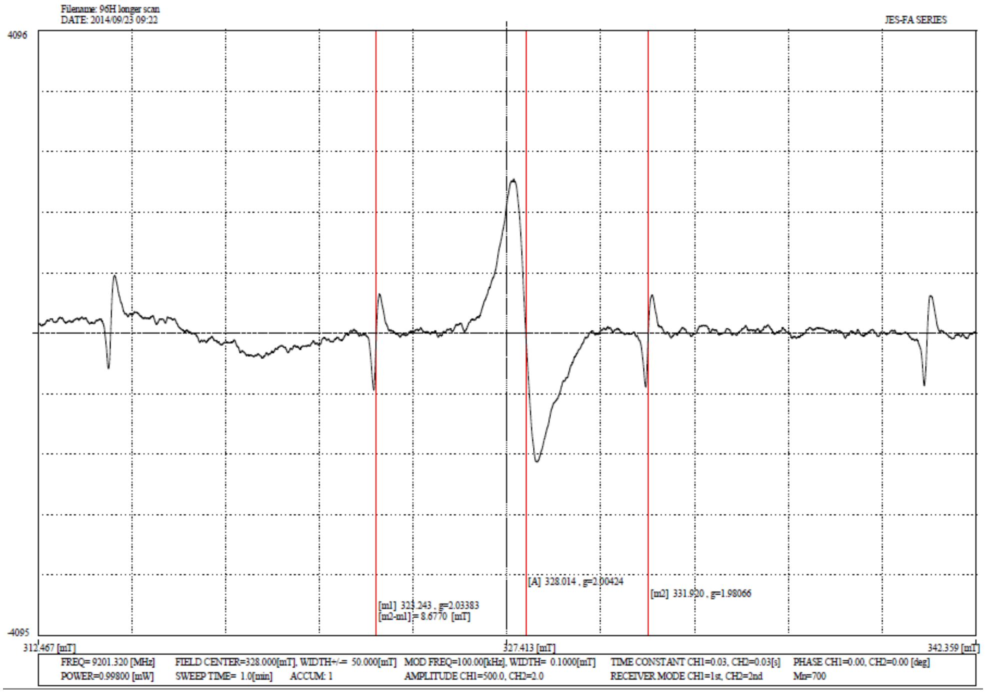

Supplementary Fig. 17: EPR of **3** showing singlet in the solid state at room temperature with *g* value = 2.00424. The additional peaks on both sides of the spectrum are due to the standard Mn^{2+} marker.

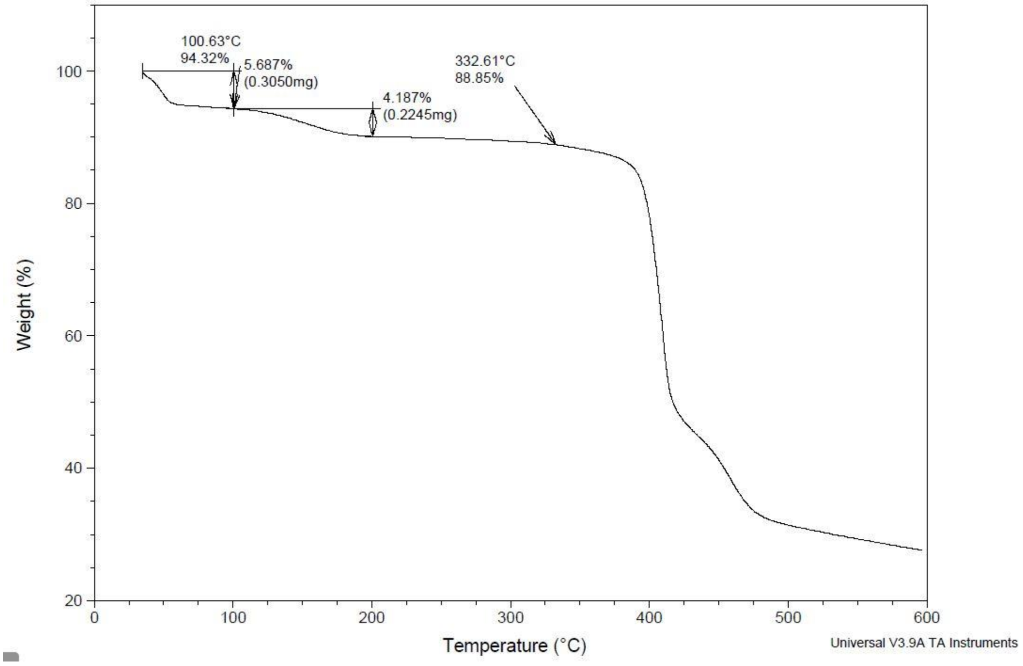

Supplementary Fig. 18: TGA of **1**. Calculated (observed) percentage weight loss of water, 5.9% (5.7%) and DMF, 5.8% (4.2%).

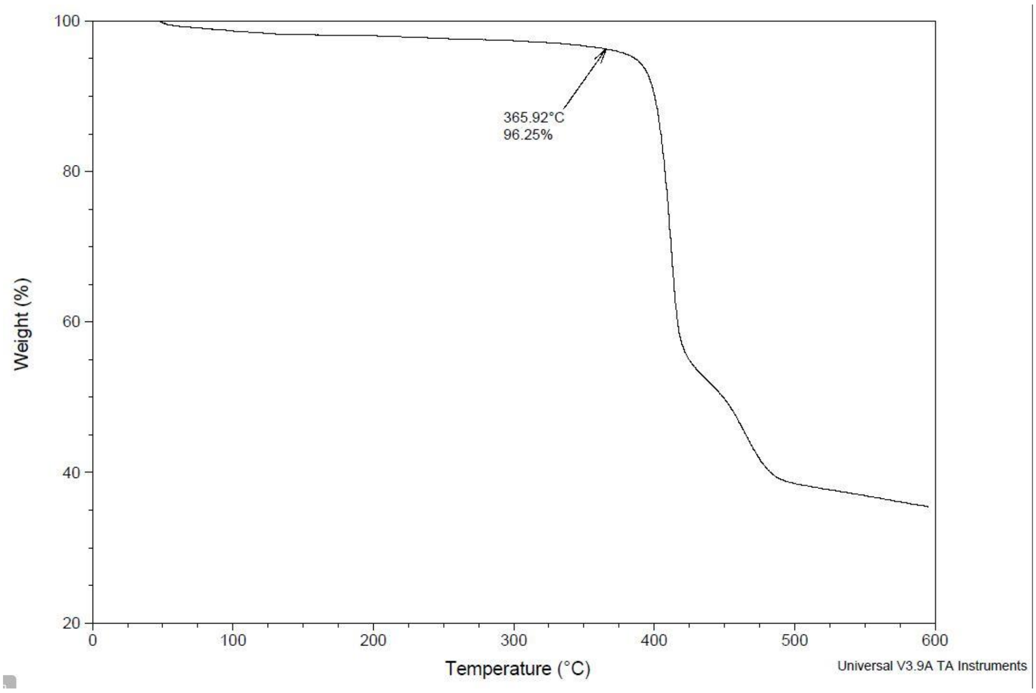

Supplementary Fig. 19: TGA of **1a**. Due to porosity of **1a**, TGA was conducted immediately after desolvation to minimize moisture absorption. However, there is an approximate 3.8% of weight loss due to absorption of water from the atmosphere.

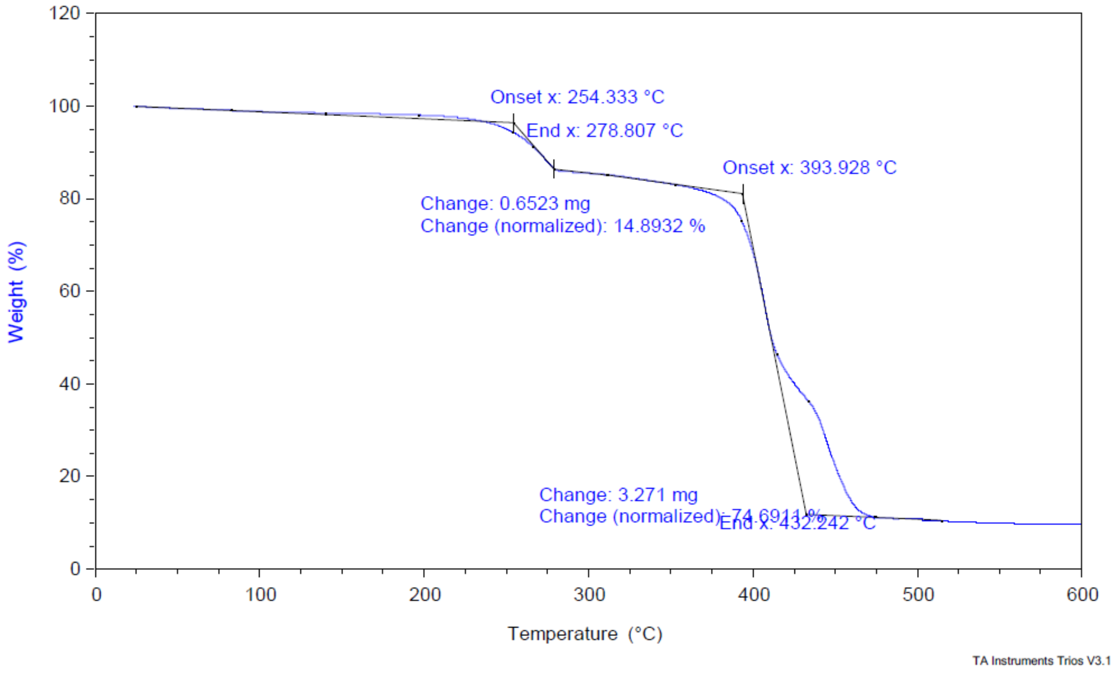

Supplementary Fig. 20: TGA of **2**. Weight loss of encapsulated perylene, melting point 276-279°C (lit.).

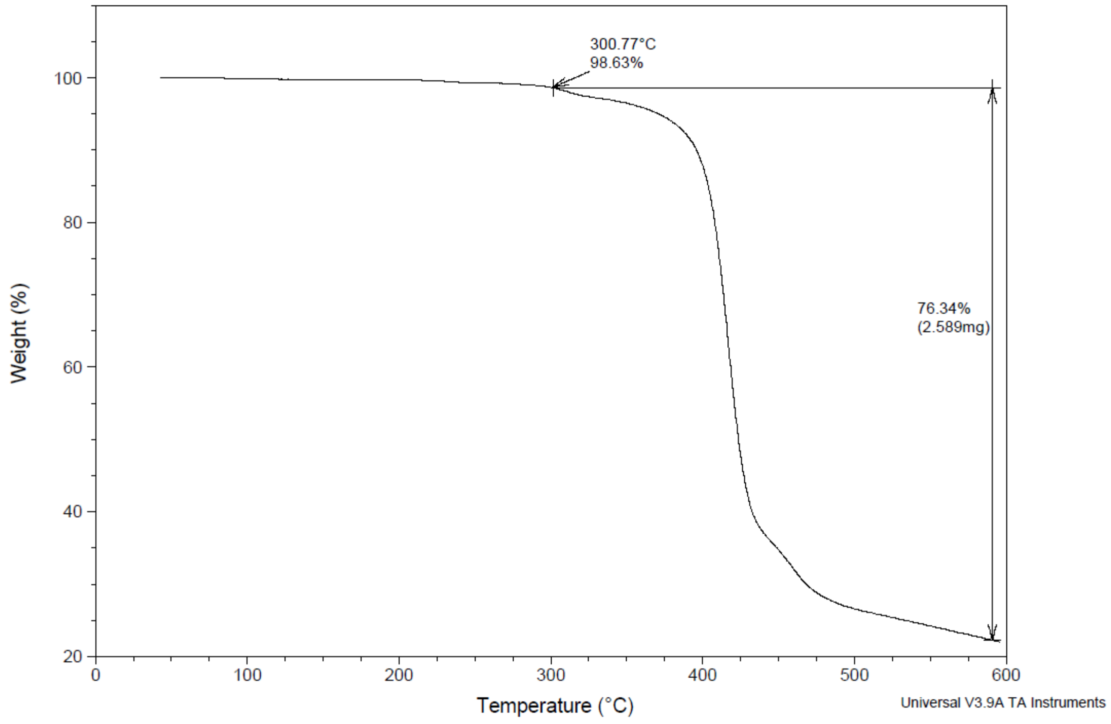

Supplementary Fig. 21: TGA of **3**.

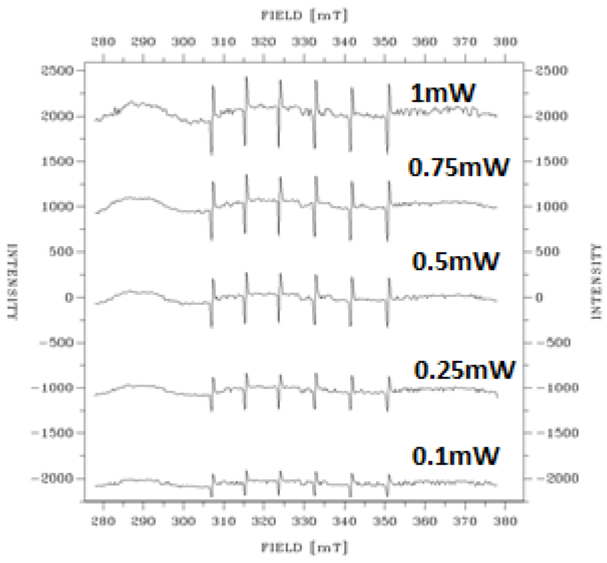

Supplementary Fig. 22: EPR of anthracene in the solid state at room temperature. There is no EPR signal at all power used. The spectrum was calibrated using the standards *g* = 2.03 and 1.98 using Mn^{2+} markers.

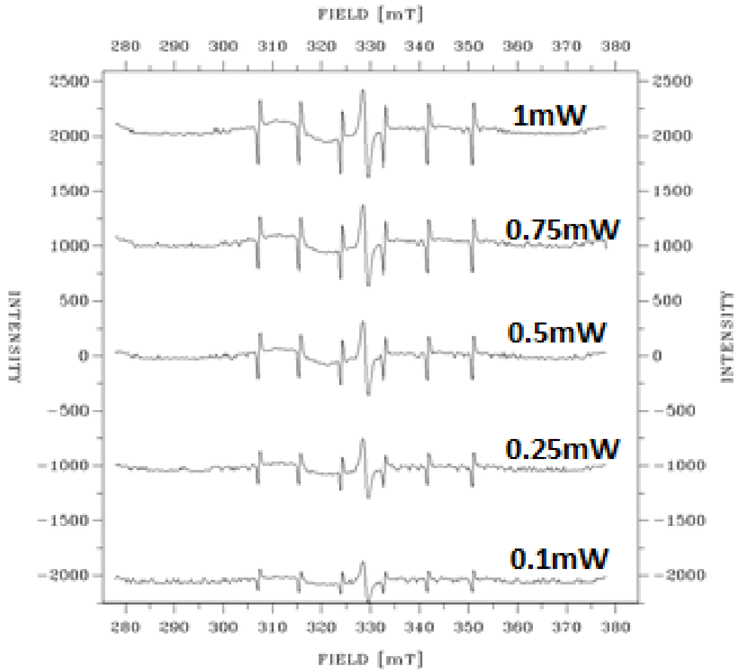

Supplementary Fig. 23: EPR of perylene showing singlet in the solid state at room temperature. A power dependence plot from 0.1mW to 1mW is shown. The spectrum was calibrated using the standards *g* = 2.03 and 1.98 using Mn^{2+} markers.

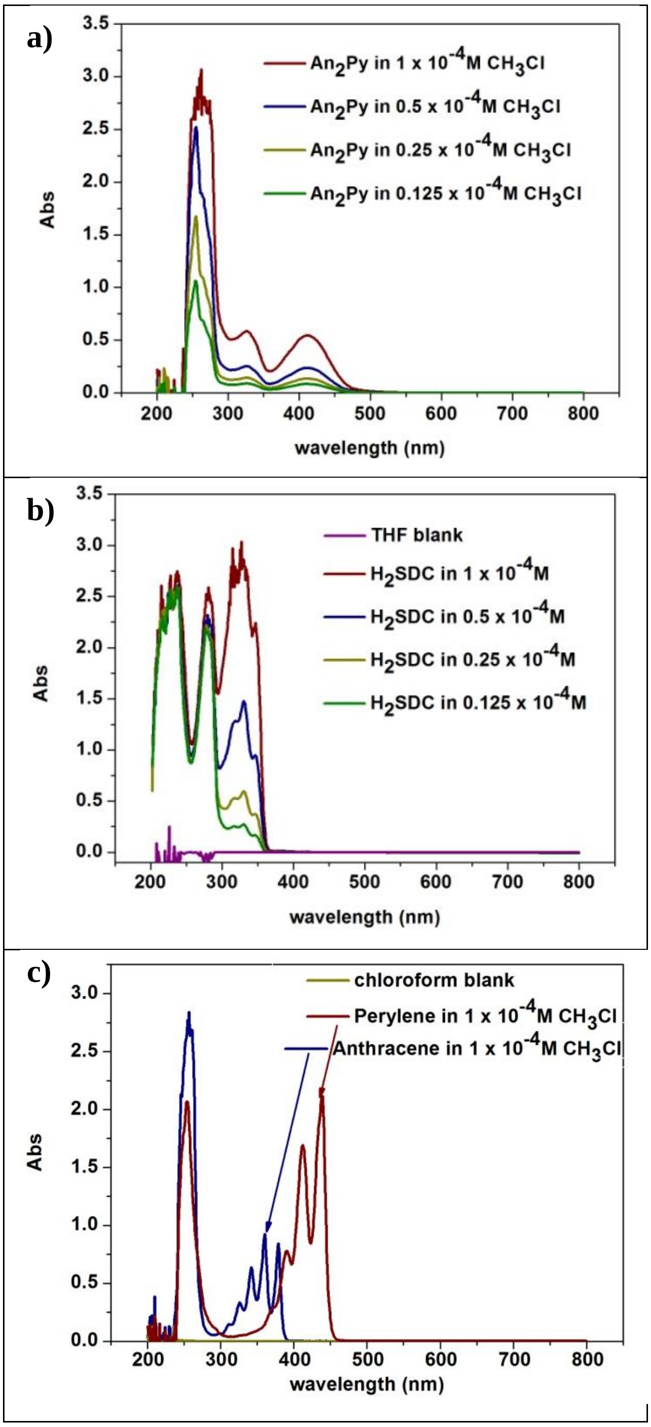

Supplementary Fig. 24: Solution photoluminescence of An2Py and H_2SDC. (**a**) Concentration variation UV-vis absorbance spectra of An2Py; (**b**) concentration variation UV-vis absorbance spectra of H_2SDC; and (**c**) solution UV-vis absorbance spectra of guests used.

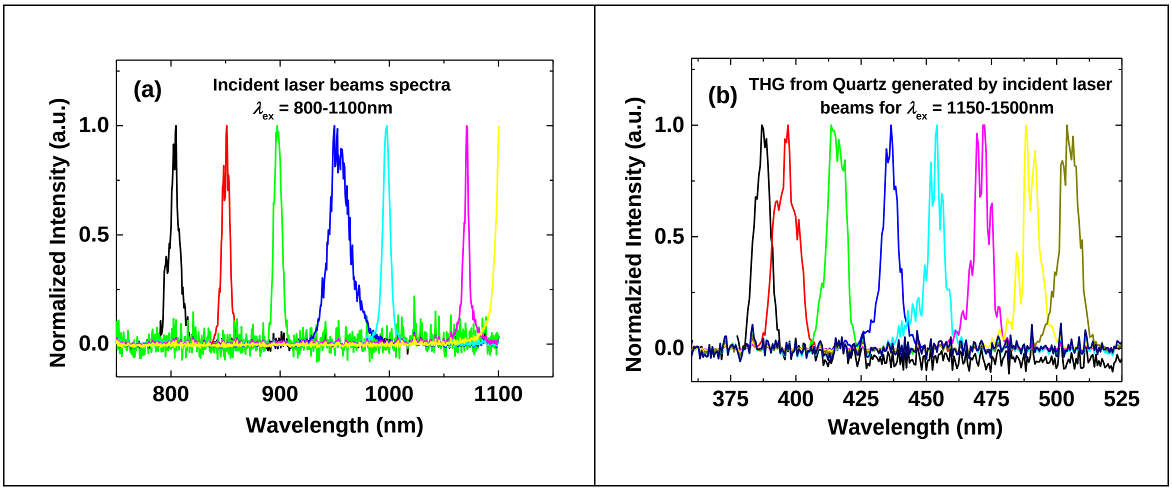

Supplementary Fig. 25: Excitation of laser source at various wavelengths and the third harmonic generation emission from quartz holder. (a) Incident laser spectra at wavelengths of 800, 850, 900, 950, 1000, 1070, and 1100 nm, and **(b)** third-harmonic generation (THG) spectral profiles of the excitation laser beams (with high intensities) passing through a 1-mm-thick quartz plate at wavelengths of 1150, 1200, 1250, 1300, 1350, 1400, 1450, and 1500 nm.

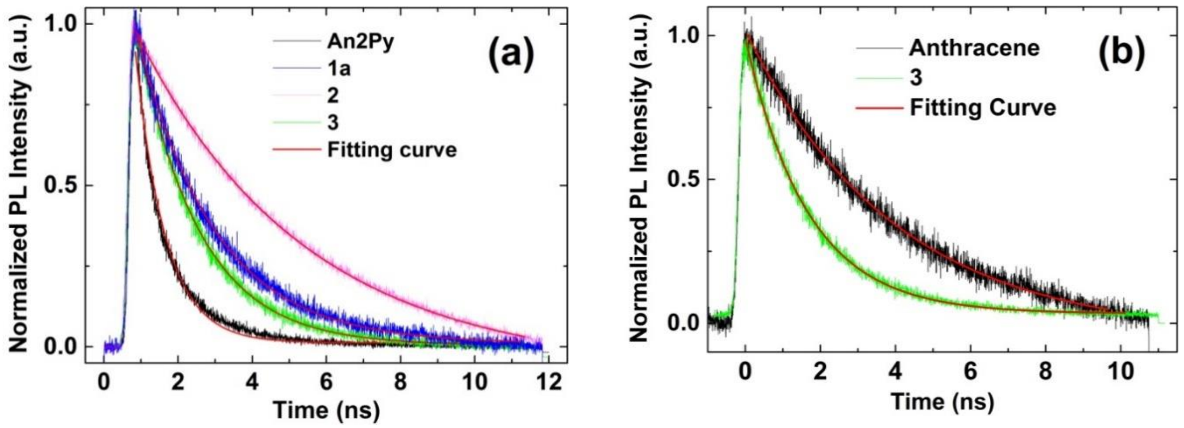

Supplementary Fig. 26: Lifetime measurements (**a**) Fluorescence decay profiles of the ligand An2Py (black), compound **1a** (blue), compound **2** (magenta) and compound **3** (green), the red curves are the fitting with monoexponential decays of 0.8-, 2.09-, 4.53- and 1.67 ns, respectively. The excitation wavelength is at 800nm, and the detection fluorescence wavelength is at 570nm. (**b**) Fluorescence decay curves of the compound 3 (green) and its guest Anthracene (black), the red curves are the fitting with monoexponential decays of 1.67 ns and 3.96 ns, respectively. The excitation and detection wavelengths for compound 3 are at 800nm and at 570 nm. For the guest Anthracene they are at 650nm and 410nm duo to its relatively different absorption and fluorescence spectra. This result indicates the FRET (Förster resonance energy transfer) efficiency in compound 3 is $E = 1- \tau_{\text{compound 3}}/\tau_{\text{Anthracene}} = 1- 1.67/3.96 \approx 58\%$.

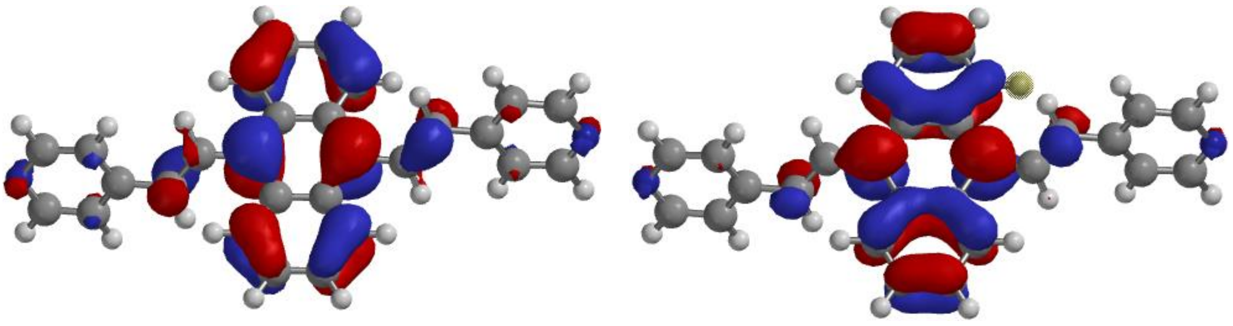

A_g A_u

Supplementary Fig. 27: The two active molecular orbitals involved in CASSCF(2,2) calculations.

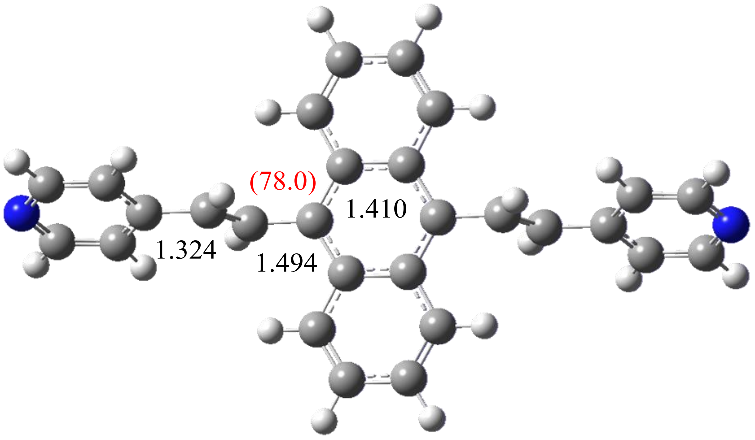

closed-shell singlet (^1A_g)

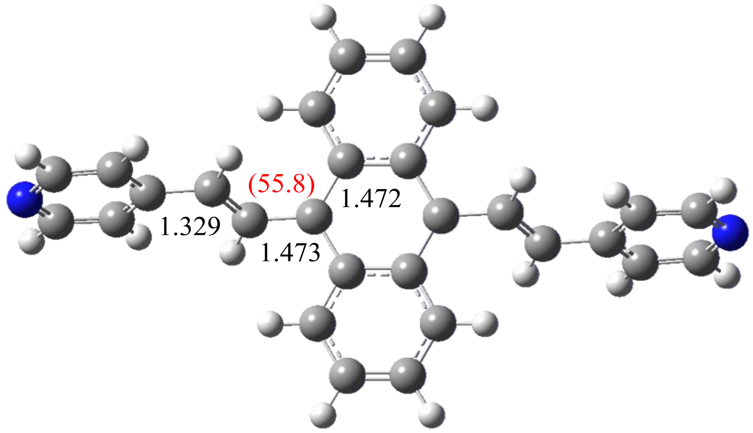

triplet (^3A_u)

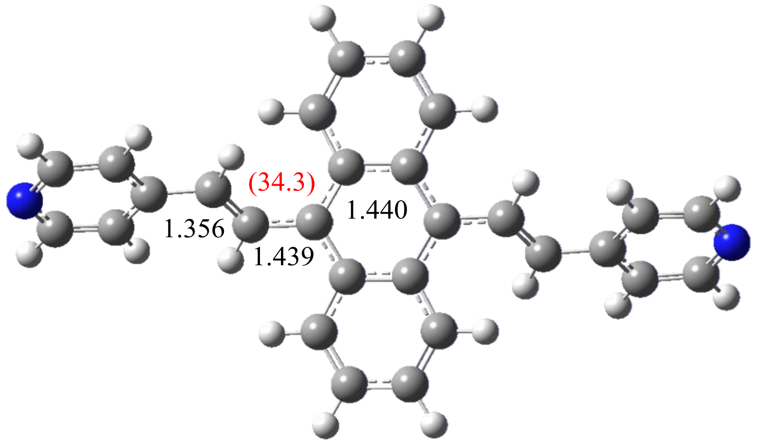

open-shell singlet (^1A_u)

Supplementary Fig. 28: Optimized (CASSCF(2,2)/6-31G*)) geometries of the three electronic states of An2Py. Bond lengths in Å and torsional angles (given in parentheses) in degrees.

Supplementary Table 1: Molar concentration calculation of the samples and standard enclosed in the cuvette used for MEPL measurements.

Samples	Weight mg	Molar Weight (g mol^{-1})	Volume (cm^3)	Molar concentration (10^{-4} mol cm^{-3})
Compound **1**	(5.4)	1229	1/2×0.6×0.6×0.1	2.4
Compound **2**	19.4	1299	1 ×0.37×0.1	4.0
Compound **3**	18.8	1225	1×0.35×0.1	4.4
An2Py	19.5	384	1×0.37×0.1	14
H_2SDC	35.5	268	1×0.85×0.1	16
Standard (Perylene)	30.1	252	1×0.4×0.1	30

Supplementary Table 2: Measurement Parameters & Data

Detector	Vantec , position sensitive detector (PSD)
PSD window	6 degrees
Soller Slits	2.5 degrees
Divergence slit	0.5 degrees
Angle Ranges	Range 1: 2 - 105 degrees (2Theta)
Step size	0.01670578 degrees (2Theta)
Time per step	Range 1: 0.5 sec<br>Total time: 57 min
Source Potential	30 kV
Source current	40 mA
Others	Sample rotation on, air scattering screen used

Supplementary Table 3: Crystal cells data

Compound	**1**	**2**	**3**
Formula	$C_{66}H_{54}N_4O_{12}Zn_2$	$C_{80}H_{52}N_2O_8Zn_2$	$C_{74}H_{50}N_2O_8Zn_2$
Formula weight	1229.87	1299.97	1225.90
Crystal system	Monoclinic	Monoclinic	Monoclinic
Space Group	*C*2/*c*	$P2_1/n$	$P2_1/n$
a, Å	15.084(13)	9.190(3)	9.3342(7)
b, Å	27.43(2)	17.557(5)	16.2033(13)
c, Å	15.799(13)	19.107(6)	19.0664(13)
β	95.447(14)°	102.375(7)°	101.826(2)°
V, $Å^3$	6508(9)	3011.1(15)	2822.5(4)
Z	4	2	2
$\rho_{cal,}$ Mg/m^3	1.255	1.434	1.442
μ, mm^{-1}	0.798	0.862	0.914
T	100(2) K	100(2) K	100(2) K
R1	0.0716	0.0532	0.0392
wR2	0.1938	0.1249	0.0785
GOF	1.083	1.037	1.025

Supplementary Discussion

Excitation intensity dependence of MEPL signal at 800, 1200 and 1500 nm; comparison between one-photon excited and MEPL spectra; and time resolved fluorescence measurements. Supplementary Fig. 11 shows the excitation intensity dependence of MEPL for all the compounds used. The slopes clearly indicate the occurrence of multi-photon absorption processes. As shown by Supplementary Fig. 12, the comparison between one-photon-excited and MEPL spectra indicates that the emission involves the same electronic state of the MOFs which is independent of the excitation wavelength. (Since the three-photon and four-photon excited PL signals are relatively weak and noisier, we have performed the average for every five sequential points for their PL spectra for better comparison.) It should be pointed out that our MEPL set up was calibrated by the measurements of third harmonic generation (THG) signals of a quartz plate, as displayed in Supplementary Fig. 25. Time-resolved measurements for the multi-photon fluorescence (two-photon shown here) were performed with the time-correlated single photon counting(TCSPC) technique utilizing femtosecond laser pulses excitation at 800 nm for An2Py, **1a**, **2**, **3** and at 650 nm for guest

molecule anthracene, as shown in Supplementary Fig. 26. The measurement results reveal that the lifetimes of the investigated up-conversion fluorescence (or of the excited states) are several nanoseconds, and the FRET (Förster resonance energy transfer) efficiency in compound **3** is $E = 1- \tau_{\text{compound 3}}/\tau_{\text{Anthracene}} = 1- 1.67/3.96 \approx 58\%$.

Computational Methods – Multi-configurational Calculations of the Three Electronics States of An2Py

There are three possible electronic states of An2Py (with C_i symmetry), namely ^3A_u, ^1A_u, and ^1A_g, arising from two electrons occupying two orbitals, namely, A_g and A_u (see Supplementary Fig. 27). We investigated the structures and energies of these three states using multi-configurational methods, which are expected to provide a proper description of the near degeneracy problem involved. Geometry optimizations were carried out with complete active space SCF (CASSCF)[10] calculations with an active space involving two electrons and two orbitals (CASSCF(2,2)), using the 6-31G* basis set. The two active orbitals involved in these CASSCF calculations are A_g and A_u (see Supplementary Fig. 27). Higher level relative energies were obtained through single-point calculations inclusion of dynamic electron correlation at second-order multi-reference perturbation (CASPT2)[11] level, based on the CASSCF(2,2)/6-31G* optimized geometries. All multi-configurational calculations were performed using the MOLPRO program[12]. The optimized geometries are shown in Figure S28. Several structural features are worthwhile to note. Firstly, the torsional angle between the anthracene moiety and the main conjugated chain is significantly different for the three electronic states. This torsional angle is significantly smaller for the open-shell singlet (34.3°), compared to those in triplet (55.8°) and closed-shell singlet (78.0°). Secondly, the olefin double bond is significantly longer for the open-shell singlet state (1.356 Å, Figure S28). Thirdly, the central ring of the anthracene moiety is less aromatic in the triplet state. Our multi-configurational calculations clearly established that the closed-shell singlet $1A_u$ state is the ground state of An2Py. Both the triplet ($3A_u$) and open-shell singlet ($1A_g$) lie significantly higher in energies, by 167.1 and 165.8 kJ/mol, respectively. Interestingly, the open-shell singlet is slightly more stable than the triplet, by just 1.3 kJ/mol. In summary, the computational result is consistent with the solution-phase experiment, which did not show any EPR signal.

Supplementary Methods

Synthesis and Structural Characterization

All the chemicals and solvents were of reagent or better grade and purchased from different commercial resources and used without further purification. An2Py and H_2SDC were synthesized according to a reported procedure[1-4]. Powder X-ray diffraction (PXRD) data were recorded on a D5005 Siemens X-ray diffractometer with graphite monochromatized Cu Kα radiation (λ = 1.54056 Å) at room temperature (298 K). ^{1}H-NMR spectra were recorded on a 300 MHz BrukerAvance 300 FT-NMR spectrometer by calibrating the residual solvent as the reference in D_6-DMSO solution. Thermogravimetric analysis (TGA) of **1**, **1a** and **3** were performed under nitrogen atmosphere with a heating rate of 5˚C min^{-1} on a TA instruments SDT-2960. Analysis was done in Universal V3.9A. TGA of **2** was obtained using Discovery TGA TA instrument under nitrogen gas flow with a heating rate of 5˚C min^{-1}, and analysis was done with Trios V3.1. The C, H, N analysis were carried using ElementarVario Micro Cube instrument at the Elemental Analysis Lab, CMMAC, Department of Chemistry, National University of Singapore. Continuous wave X-band EPR spectra were obtained with a JEOL JES-FA200 EPR spectrometer using MgO:Mn^{2+} marker The Mn marker consists of six Mn^{2+} signals which the third (2.034) and fourth (1.981) signals are calibrated to account for field variations.

PXRD measurement for desolvated compound 1:

The sample amount was too small for a large sample holder. A small amount of the sample was dispersed onto a low background sample holder (Si-crystal cut along (011) planes) and measured on a Bruker D8 diffractometer (Bruker-AXS GmbH, Karlsruhe, Germany) equipped with a Cu-Ka source, 2.5 degree soller slits, 0.5 degrees divergence slit, an air scattering screen and a gas-filled position sensitive detector. Further details are in the table below:

TOPAS and Rietveld refinements

Rietveld refinements with Topas

Rietveld refinements were performed with Topas v4.2 (Bruker-AXS GmbH, Karlsruhe, Germany). The main phase was D708 (desolvated compound 1). The analysis clearly showed

that the symmetry was lowered to the triclinic space group P-1. Rigid body formalism was used to describe the movement of the large linker molecules.

Preparation of the ligand An2Py: The 3 steps synthesis was according to reported procedures[2-4]. They are briefly mentioned below.

1) 1.78 g of anthracene was mixed with 3.56 g N-Bromosuccinimide, 0.16 g $LiClO_4$ and 0.64 g SiO_2 in 100 ml dichloromethane for 30 mins. The resulting yellow precipitate was filtered off and filtrate was washed with copious amount of water via separatory funnel. The washed organic layer was allowed to crystallize. Yellow needle-like crystals (9,10 dibromoanthracene-$AnBr_2$) were formed. Yield = 99%.

2) 2.004 g of $AnBr_2$ were mixed with 8.1 ml of 1.6 M *n*BuLi in dry ether at 0˚C for 2 h. To the resulting orange mixture was added dry dimethylformamide (DMF) (4.5 ml) at -78˚C. The entire reaction was allowed to proceed for about 10 h before working up with water. The orange precipitate (9,10-anthracenedicarbaldehyde-$AnAd_2$) that ensued was filtered and used for the next step. Yield = 30%.

3) 0.468 g of $AnAd_2$ was refluxed with 4.4 ml of 4-picoline in 5 ml of acetic anhydride for 12 h at 100˚C. The mixture was allowed to stir at room temperature for another 12 h. The yellow precipitate that was filtered off was recrystallized from DMF. Yellow blocky crystals (An2Py) were formed. Yield = 30%.

Preparation of $[Zn_2(\textit{SDC})_2(An2Py)]\cdot DMF\cdot 4H_2O$ (1): 9.3 mg (0.025 mmol) of $Zn(ClO_4)_2$, 4.8 mg (0.0125 mmol) of An2Py and 6.7 mg (0.025 mmol) of H_2SDC were weighed in a 25 ml scintillating vial. 2 ml of DMF and 1ml of water was added to the solids with a drop of HNO_3. The bright red solution that formed in the vial was capped and heated in the oven for 2 days at 120°C followed by slow cooling. Bright yellow plate-like crystals were obtained and washed with DMF. Yield: 50%. ^1H NMR (DMSO, D_6, 300 MHz): An2Py ligand: δ8.97 (d, 4H, aromatic proton), δ8.95 (d, 2H, vinyl proton), δ8.50 (d, 4H, aromatic proton), δ8.42 (m, 4H, aromatic-anthracene proton), δ7.65 (m, 4H, aromatic-anthracene proton), δ7.30 (d, 2H, vinyl proton). SDC ligand: δ7.93 (d, 4H, aromatic proton), δ7.75 (d, 4H, aromatic proton), δ7.47 (s, 2H, vinyl proton). Elemental analysis (%) Calcd for C-68.83, H-3.82, N-2.68 Found C-69.04, H-3.95, N-2.34 for the desolvated compound.

Preparation of [Zn_2(*SDC*)$_2$(An2Py)] (1a): (**1**) was ground and heated at 100˚C for 2 h under vacuum. The bright yellow powder was used for further characterization. Yield: 99%. ^{1}H NMR (DMSO, D_6, 300MHz): An2Py ligand: δ8.97 (d, 4H, aromatic proton), δ8.95 (d, 2H, vinyl proton), δ8.50 (d, 4H, aromatic proton), δ8.42 (m, 4H, aromatic-anthracene proton), δ7.65 (m, 4H, aromatic-anthracene proton), δ7.30 (d, 2H, vinyl proton). SDC ligand: δ7.93 (d, 4H, aromatic proton), δ7.75 (d, 4H, aromatic proton), δ7.47 (s, 2H, vinyl proton).

Preparation of [Zn_2(*SDC*)$_2$(An2Py)(perylene)] (2): 9.3 mg (0.025 mmol) of $Zn(ClO_4)_2$, 4.8mg (0.0125 mmol) of An2Py, 6.7 mg (0.025mmol) of H_2SDC and 3.15 mg (0.0125 mmol) of perylene were weighed in a 25 ml scintillating vial. 2ml of dimethyl formamide and 1ml of water was added to the solids with a drop of HNO_3. The bright red solution that formed in the vial was capped and heated in the oven for 2 days at 120°C followed by slow cooling. Bright orange plate-like crystals were obtained and washed with DMF. Yield: 53%. ^{1}H NMR (DMSO, D_6, 300MHz): An2Py ligand: δ8.97 (d, 4H, aromatic proton), δ8.95 (d, 2H, vinyl proton), δ8.50 (d, 4H, aromatic proton), δ8.42 (m, 4H, aromatic-anthracene proton), δ7.65 (m, 4H, aromatic-anthracene proton), δ7.30 (d, 2H, vinyl proton). SDC ligand: δ7.93 (d, 4H, aromatic proton), δ7.75 (d, 4H, aromatic proton), δ7.47 (s, 2H, vinyl proton). Perylene guest: δ8.34 (d, 4H, aromatic-perylene proton), δ7.78 (d, 4H, aromatic-perylene proton), δ7.53 (t, 2H, aromatic-perylene proton). Elemental analysis (%)Calcd for C-73.90, H-4.00, N-2.16 Found C-72.36, H-4.02, N-2.35.

Preparation of [Zn_2(*SDC*)$_2$(An2Py)(anthracene)] (3): 9.3 mg (0.025 mmol) of $Zn(ClO_4)_2$, 4.8 mg (0.0125 mmol) of An2Py, 6.7 mg (0.025 mmol) of H_2SDC and 2.2 mg (0.0125 mmol) of anthracene were weighed in a 25 ml scintillating vial. 2 ml of DMF and 1 ml of water was added to the solids with a drop of HNO_3. The bright red solution that formed in the vial was capped and heated in the oven for 2 days at 120°C followed by slow cooling. Bright yellow plate-like crystals were obtained and washed with DMF. Yield: 54%. ^{1}H NMR (DMSO, D_6, 300MHz): An2Py ligand: δ8.97 (d, 4H, aromatic proton), δ8.95 (d, 2H, vinyl proton), δ8.50 (d, 4H, aromatic proton), δ8.42 (m, 4H, aromatic-anthracene proton), δ7.65 (m, 4H, aromatic-anthracene proton), δ7.30 (d, 2H, vinyl proton). SDC ligand: δ7.93 (d, 4H, aromatic proton), δ7.75 (d, 4H, aromatic proton), δ7.47 (s, 2H, vinyl proton). Anthracene guest: δ8.57 (s, 2H, aromatic proton), δ8.07 (m, 4H, aromatic-anthracene proton), δ7.50 (m, 4H, aromatic-

anthracene proton). Elemental analysis (%) Calcd for C-72.49, H-4.08, N-2.28 Found C-70.94, H-4.12, N-2.48.

CCDC 1041092-1041094 contains the supplementary crystallographic data for this paper. These data can be obtained free of charge from the Cambridge Crystallographic Data Centre via www.ccdc.cam.ac.uk/data_request/cif.

Optical characterization with one-photon excitation

Solution samples were diluted to 10^{-3} M or 10^{-4} M and the UV-vis absorption spectra was measured on UV-1601 Shimadzu UV-visible spectrometer. Solution fluorescence intensity measurements were performed using the Cary Eclipse Spectrophotometer with Cary WinFLR software at 350 nm excitation wavelength. Solid-state diffuse reflectance UV-vis spectra were measured on UV-2450 Shimadzu UV-visible spectrometer equipped with an integrating sphere and barium sulphate as reflecting reference. The solid-state absolute fluorescence quantum yields of the An2Py and **1a**, **2**, **3** were measured by a steady-state and phosphorescence lifetime spectrometer (FSP920, Edinburgh) coupled with an integrating sphere (150 mm; internally coated with barium sulphate) with the excitation at 400 nm. Using the same instrumental setup, the solution quantum efficiencies of An2Py (excited at 400 nm) and H_2SDC (excited at 350 nm) were measured. All the measurements are shown in Supplementary Fig. 6-9, 24.

Optical characterization with multi-photon excitation

The multi-photon excited photoluminescence (MEPL) measurements were performed at room temperature in the wavelength range of 800-1500 nm. In these measurements, the excitation laser pulses (1 kHz, 285-2600 nm, pulse width < 150 fs) were generated by an optical parametric amplifier (TOPAS-C, Light-Conversion) pumped by a regenerative amplified femtosecond Ti:sapphire laser system (800nm, 1 kHz, pulse energy 3 mJ, pulse width < 150 fs, Libra, Coherent), which was seeded by a femtosecond Ti-saphhire oscillator (80 MHz, pulse width < 100 fs, 800 nm, Vitesse™ 800-2, Coherent). The frequency up-converted PL spectra and THG of the excitation pulses were recorded with a spectrometer (Avaspec-2048-SPU, Resolution of 0.5 nm). The laser power was measured by an optical

power meter (Optical power meter 1917-R, Newport) with the appropriate detector (Detector 919P-003-10, Newport).

Mostly, MEPL are measured for solution samples contained in a 1-cm-thick quartz cuvette, where the incident laser beam can be focused into the middle part with small loss and the MEPL was collected in the perpendicular direction to the excitation beam. Different from this, our samples were compact solid powder and the interaction between incident laser beams and the powder samples mainly occurred on the surface with small interaction length. Therefore, we applied a slightly different experimental setup for measuring the MEPL of our powder samples, as illustrated in Supplementary Fig. 10. The incident laser pulses were focused by a lens (L1) with a focal length of 6 cm. The distance between the powder samples contained in a 1-mm-thick quartz cuvette and L1 was kept at 7.5 cm, in order to avoid the high excitation peak intensity on the samples. The spot size of the incident laser beam was controlled at 3 mm by the iris before L1. Incident angle between the incident laser beam and the sample plane was maintained at 45°, and MEPL signal was collected in the perpendicular direction of the incident light using a collection system of two 10 cm focal length lenses (L2 and L3). The excitation laser beams were filtered out by a short-pass filter cut at 750 nm and the obtained pure MEPL signal was coupled into a spectrometer (Avaspec-2048-SPU, Resolution of 0.5 nm). As a calibration, a standard solid state sample, well grinded perylene single crystal, which shows two-photon excited fluorescence at 800 nm[5-6], was employed in the same experimental set-up. Although our experimental set-up is slightly different from the normal one for solution samples, the applied relative measurement method utilizing a standard sample can give us reasonable and accurate results.

Determination of the multi-photon absorption action cross-sections of the investigated samples.

MEPL strength F_n can be obtained by integrating Δf_n over the entire laser focused volume and time. Δf_n is given by[7-9]:

$$\Delta f_n = (1/n)\emptyset\eta\sigma_n\rho I_{\mathrm{r}}^n \,\mathrm{d}s\,\mathrm{d}z\,\mathrm{d}t\,/(\hbar\omega)^n \qquad (1)$$

The factor of $1/n$ accounts for the fact that n photons are absorbed from the near infrared (NIR) laser light for each fluorophore excitation generated. $\hbar\omega$ is the photon energy of the NIR incident laser beam. $\emptyset$ is the fluorescence collection efficiency of the experimental setup,

η is the PL quantum yield, σ_n is the multiphoton absorption cross-section, ρ is the sample molar concentration, ds dz is the small volume of the focused laser beam considered, and I_r is the nearly constant laser intensity at this small volume.

In our case, the sample is compact solid powder and the interaction length between incident laser beams and the sample will be small, therefore, we can reasonably assume this interaction length as small constant L_0 and hence, Eq. (1) approximates to

$$\Delta f_n = (1/n)\emptyset\eta\sigma_n\rho I_r^n L_0 \, \mathrm{d}s \, \mathrm{d}t \, /(\hbar w)^n \qquad (2).$$

Since the spatial profiles of the input laser pulses are Gaussian functions, the integration out of the beam radius region at the sample position are relative small compared to that within the beam radius and can be neglected. Under these approximations and with the assumption that the temporal profiles of the input laser pulses are also Gaussian function, the totally collected MEPL signal can be obtained by performing integration for (2) as follows.

By defining that the directions of x and y axes are indicated as in the following diagram (y is in the direction perpendicular to the plane of paper and outward, O is the zero point), we have the laser beam intensity as:

$$I_r = I_{00}\left[1+\left((d+x\cos(\pi/4))/z_0\right)^2\right]^{-1}\exp\left[\frac{-2x^2-2y^2}{w_0^2\left[1+\left((d+x\cos(\pi/4))/z_0\right)^2\right]}-\frac{t^2}{\tau^2}\right]$$

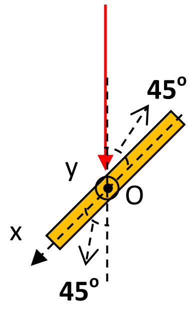

where τ is the pulse duration of the Gaussian laser pulse, I_{00} is the peak intensity of the input laser pulse at the focal point, ω_0 is the beam waist and z_0 is the diffraction length and d is the distance between the focal point of laser beam and the central point on the sample.

In order to determine the two-photon absorption (2PA) excited fluorescence strength, we have

$$F_2 = \frac{\int_{-\infty}^{+\infty}\int_{-\infty}^{+\infty}\int_{-\infty}^{+\infty} \phi\eta\sigma_2\rho L_0 I_r^2 \, dx\, dy\, dt}{2(\hbar\omega)^2}$$

$$= \phi\eta\sigma_2\rho L_0 I_{00}^2 w_0 \frac{\tau\pi}{\sqrt{2}} \frac{1}{4(\hbar\omega)^2} \int_{-\infty}^{+\infty} \frac{1}{\left[1+\left((d+x\cos(\pi/4))/z_0\right)^2\right]^{3/2}} \exp\left[\frac{-4x^2}{w_0^2\left[1+\left((d+x\cos(\pi/4))/z_0\right)^2\right]}\right] dx$$

$$\approx \phi\eta\sigma_2\rho L_0 I_{00}^2 w_0 \frac{\tau\pi}{\sqrt{2}} \frac{1}{4(\hbar\omega)^2} \int_{-\infty}^{+\infty} \frac{1}{(d/z_0)^3} \exp\left[\frac{-4x^2}{w_0^2 (d/z_0)^2}\right] dx$$

$$\approx \frac{\pi^{3/2}}{8\sqrt{2}(\hbar\omega)^2} \tau\phi\eta\sigma_2\rho L_0 I_{00}^2 w_0^2 \left(\frac{z_0}{d}\right)^2$$

Similarly, we can obtain the expressions of three-photon and four-photon absorption (3PA and 4PA) excited fluorescence strength:

$$F_3 = \frac{\pi^{3/2}}{18\sqrt{3}(\hbar\omega)^3} \tau\phi\eta\sigma_3\rho L_0 I_{00}^3 w_0^2 \left(\frac{z_0}{d}\right)^4$$

$$F_4 = \frac{\pi^{3/2}}{64(\hbar\omega)^4} \tau\phi\eta\sigma_4\rho L_0 I_{00}^4 w_0^2 \left(\frac{z_0}{d}\right)^6$$

In order to determine the multi-photon action cross-section of the investigated samples, two-photon PL of a standard sample, perylene in solid state (powder) was measured at 800 nm utilizing the same experimental setup. Two-photon absorption cross-section of perylene is reported to be 3.0 GM ($3 \times 10^{-50} \mathrm{cm}^4 \cdot \mathrm{s} \cdot \mathrm{photon}^{-1}$) at 800 nm and its quantum yield at solid state (powder) is ~ 0.18.[5-6] Two-photon action cross-sections of the our samples at 800 nm can be obtained from the ratio of the measured PL strengths from the perylene to the samples $(F_{2(\mathrm{Py})}/F_{2(\mathrm{x})}) = [(\eta\sigma_2)_{\mathrm{Py}} \cdot \rho_{\mathrm{Py}} \cdot (I_{00}^2)_{\mathrm{Py}}]/[(\eta\sigma_2)_{\mathrm{x}} \cdot \rho_{\mathrm{x}} \cdot (I_{00}^2)_{\mathrm{x}}]$, where $F_{2(\mathrm{Py})}$ and $F_{2(\mathrm{x})}$ are the measured PL strengths. Similarly, 3PA action cross-sections of the investigated samples at different wavelengths can be obtained from the ratio of$(F_{2(\mathrm{x})800\mathrm{nm}}/F_{3(\mathrm{x})\lambda 2}) = \{9\sqrt{3}\hbar w[(\eta\sigma_2) \cdot (I_{00}^2)\omega_0^2 z_0^2]_{\mathrm{x}(800\mathrm{nm})}\}/\{4\sqrt{2}[(\eta\sigma_3) \cdot (I_{00}^3)\omega_0^2 z_0^4/d^2]_{\mathrm{x}(\lambda 2)}\}$, where $F_{2(\mathrm{x})800\mathrm{nm}}$ is the two-photon excited PL strength measured at 800 nm and $F_{3(\mathrm{x})\lambda 2}$is the three-photon excited PL strength at λ_2 . 4PA action cross-sections of the samples could be determined from the ratio between the four-photon excited PL strength at λ_3 and the two-photon excited PL

strength at 800 nm, as: $(F_{2(\mathrm{x})800\mathrm{nm}}/F_{4(\mathrm{x})\lambda 3}) = 4\sqrt{2}(\hbar w)^2[(\eta\sigma_2)\cdot(I_{00}^2)\omega_0^2 z_0^2]_{\mathrm{x}(800\mathrm{nm})}/[(\eta\sigma_4)\cdot(I_{00}^4)\omega_0^2 z_0^6/d^4]_{\mathrm{x}(\lambda 3)}$.

By applying the various experimental parameters (including the molar concentrations of different samples as shown in Table S3) into the above expressions, 2PA, 3PA and 4PA action cross-sections of the investigated samples can be determined.

Supplementary References

1. Toland, W. G., Wilkes, J. B. & Brutschy, F. J. Reactions of Toluic Acids with Sulfur. I. Stilbenedicarboxylic Acids. *J. Am. Chem. Soc.* **75**, 2263-2264 (1953).

2. Mojtaba, B., Najmedin, A. &. Saidi, M. R An Intriguing effect of lithium perchlorate dispersed on silica gel in the bromination of aromatic compounds by N-bromosuccinidmide. *Canad. J. Chem.* **83**, 146-149 (2005).

3. Amanda E. Lee, Michael R. Grace, Adam G. Meyer, Kellie L. Tuck Fluorescent Zn^{2+} chemosensors, functional in aqueous solution under environmentally relevant conditions. *Tetrahed. Lett.* **51**, 1161–1165 (2010).

4. E. M. Vernigor et al. Synthesis and spectral luminescent properties of certain pyridine and quinoline analogs of isomeric distyrlnapthalenes and styryl- and distyrlanthracenes. *Chem. Heterocycl. Compds*. **23**, 677 – 680 (1987).

5. Makarov, N. S., Drobizhev, M. & Rebane, A. Two-photon absorption standards in the 550-1600 nm excitation wavelength range. *Opt. Express* **16**, 4029-4047 (2008).

6. Katoh, R., Suzuki, K., Furube, A., Kotani, M. & Tokumaru, K. Fluorescence Quantum Yield of Aromatic Hydrocarbon Crystals. *J. Phys. Chem. C.* **113**, 2961-2965 (2009).

7. Xu, C. & Webb, W. W. Measurement of two-photon excitation cross sections of molecular fluorophores with data from 690 to 1050nm. *J. Opt. Soc. Am. B* **13**, 481-491 (1996).

8. Maiti, S., Shear, J. B., Williams, R. M., Zipfel, W. R. & W. W. Webb. Measuring serotonin distribution in live cells with three-photon excitation. *Science* **275**, 530-532 (1997).

9. Xing, G., Ji, W., Zheng, Y. & Ying, Y. J. High efficiency and nearly cubic power dependence of below-band-edge photoluminescence in Water-soluble, Copper doped ZnSe/ZnS Quantum dots. *Opt. Express* **16**, 5715-5720 (2008).

10. Roos, B. O. & Lawley, K. P. The complete active space self-consistent field method and its applications in electronic structure calculations. *Adv. Chem. Phys.* **69**, 399-446 (1987).

11. Celani, P. & Werner, H.-J. Multireference perturbation theory for large restricted and selected active space reference wave functions. *Mol. Phys.* **89**, 645-661 (1996).

12. Werner H.-J. & Knowles P.J. MOLPRO, Version 2008.1, University of Birmingham (2008).
